# Methods to derive composite indicators used for quality and safety measurement and monitoring in healthcare: a scoping review

**DOI:** 10.3389/frhs.2026.1852718

**Published:** 2026-08-04

**Authors:** Thérèse McDonnell, Jaspreet Kaur Dullat, Kathleen McDonnell, Triona McNicholas, Ann Marie Murray, Marcella O’Dowd, Eilish McAuliffe

**Affiliations:** 1 IRIS Centre, School of Nursing, Midwifery & Health Systems, University College Dublin, Dublin, Ireland; 2 National Quality and Patient Safety Directorate, Health Service Executive, Dublin, Ireland

**Keywords:** composite indicator, composite measure, healthcare, quality, safety

## Abstract

**Introduction:**

The use of composite measures of quality in healthcare is widespread and designing such measures involves many technical decisions. Their use can be controversial due to concerns around transparency, appropriateness and uncertainty. This scoping review identified the methodologies adopted and methods used to derive composite measures of quality in healthcare and presents a toolbox for their development.

**Methods:**

PRISMA-ScR guidelines were followed and five electronic databases and grey literature searched. Data was extracted on 11 stages of development, adapting a framework developed by the European Commission Joint Research Centre. A scoring scheme identified publications with significant information on stages of development.

**Results:**

Methodology publications (n = 65) offered technical guidance and raised concerns about aspects of the development process. Publications developing composite measures (n = 325) presented varying levels of information. Most scored well on measure selection, data analysis, deconstruction, and presentation. However, information scores for the use of theoretical frameworks, treatment of missing data and uncertainty analysis were low.

**Conclusion:**

Checklists developed from this review provide an actionable framework to guide composite measure development in a robust and transparent manner. Examples of best practice support the use of these checklists, assisting those developing and interpreting composite measures.

**Systematic Review Registration:**

https://bmjopen.bmj.com/content/13/7/e071382.long.

## 1 Introduction

Since the Institute of Medicine (IOM)’ publication of two landmark documents on health care quality, there has been an increased focus on capturing measures of quality (1, 2). Quality measurement is commonplace across healthcare contexts internationally. Single indicators measuring a particular aspect of quality are of interest, however, research suggests that individuals faced with too many factors in making a decision take cognitive shortcuts that might not be in their best interest (3). Therefore, the multitude of indicators available may make it challenging to assess overall performance across multiple dimensions.

Composite measures are developed and used to assess the quality of healthcare in a variety of settings and specialisms, at local, national and international level. A composite measure is a combination of two or more component measures, each of which individually reflects quality of care, into a single performance measure with a single score (4). Measures can summarize quality across multiple indicators, improve ability to detect quality differences, identify important drivers of quality, help prioritize action for quality improvement, and support decisions about future health care needs (3). However, composite indicators of quality and safety within healthcare can be controversial, particularly when used to rank healthcare providers (5, 6). The summarised nature of composite measures may obscure either serious failings or excellent performance on specific elements of performance (3, 7).

It is important that composite measures developed are accurate and appropriate to what is being measured to ensure users trust and respond appropriately to the information generated. While composite measures are often criticised for the lack of specificity or their unfairness, particularly measures designed to rank and reimburse healthcare providers (5, 6), the methods used to develop these measures and the methodological concerns raised can provide rich learning for the development of all composite measures across healthcare areas. Barclay et al. declare that most flaws can be addressed, reiterating the need for greater transparency around the goal of a composite indicator, but also suggest that guidelines on the design, development and reporting of such indicators would be beneficial (8).

This scoping review aims to help improve the development of composite indicators by gathering detailed information on the development stages that need appropriate consideration if the accuracy and usefulness of a composite measure is to be assured. Adapting the 10-stage methodology for constructing composite indicators developed by the European Commission Joint Research Center (EC-JRC) (9) facilitated a structured assessment of the methodological approaches and the methods used by studies developing composite measures of quality of health care delivery (10). The adoption of an information score facilitated the identification of best practice and the development of an actionable checklist to support a two phased approach to the development of composite measures in healthcare:

- Phase I - composite measure development
- Phase II - composite measure assessment, presentation and review.

## 2 Methods

As the purpose of this review was to assess methods used to derive composite measures of quality and safety in healthcare across an extensive literature, identifying and analysing gaps and best practice, a scoping review was deemed the most suitable methodological approach (11, 12). The JBI Manual for Evidence Synthesis guided this review, and Alexander *et al's* guidance on planning and completion of large scoping reviews helped ensure methodological rigor (13, 14).

### 2.1 Eligibility criteria, information sources and search

This review was completed in line with the published protocol (15). There was no restriction on the inclusion of study types. Published peer-reviewed and grey literature publications in English, including discussion, policy, commentary, and guidance publications were included if they discussed any aspect of the stages of development of a relevant composite indicator. This review is limited to publications published from 1 January 2000 to 28th February 2025, consistent with the increased focus on quality and safety measurement in healthcare following the publication of *To Err is Human (*1).

The bibliographic database searching applied a three-step strategy (16), with the full search and screening strategy outlined in the published protocol. Following initial test searches in PubMed and Cinahl and review by an information specialist, databases were searched on 16th January 2023: PubMed, Cinahl and Embase, ABI/Inform and Safetylit. A further search update to 28th February 2025 was carried out on 10th November 2025. Grey literature searching was also conducted using Grey Literature Report, DANS EASY, Google Scholar, OpenAIRE and Overton, with direct searches of relevant websites conducted at this time. The online machine learning tool, Elicit™ (an AI research assistant), was used to search for any relevant papers missed by the search of these 5 databases (17, 18). Full search criteria are detailed in Supplementary File 1.

### 2.2 Study screening and selection

**Figure 1:**
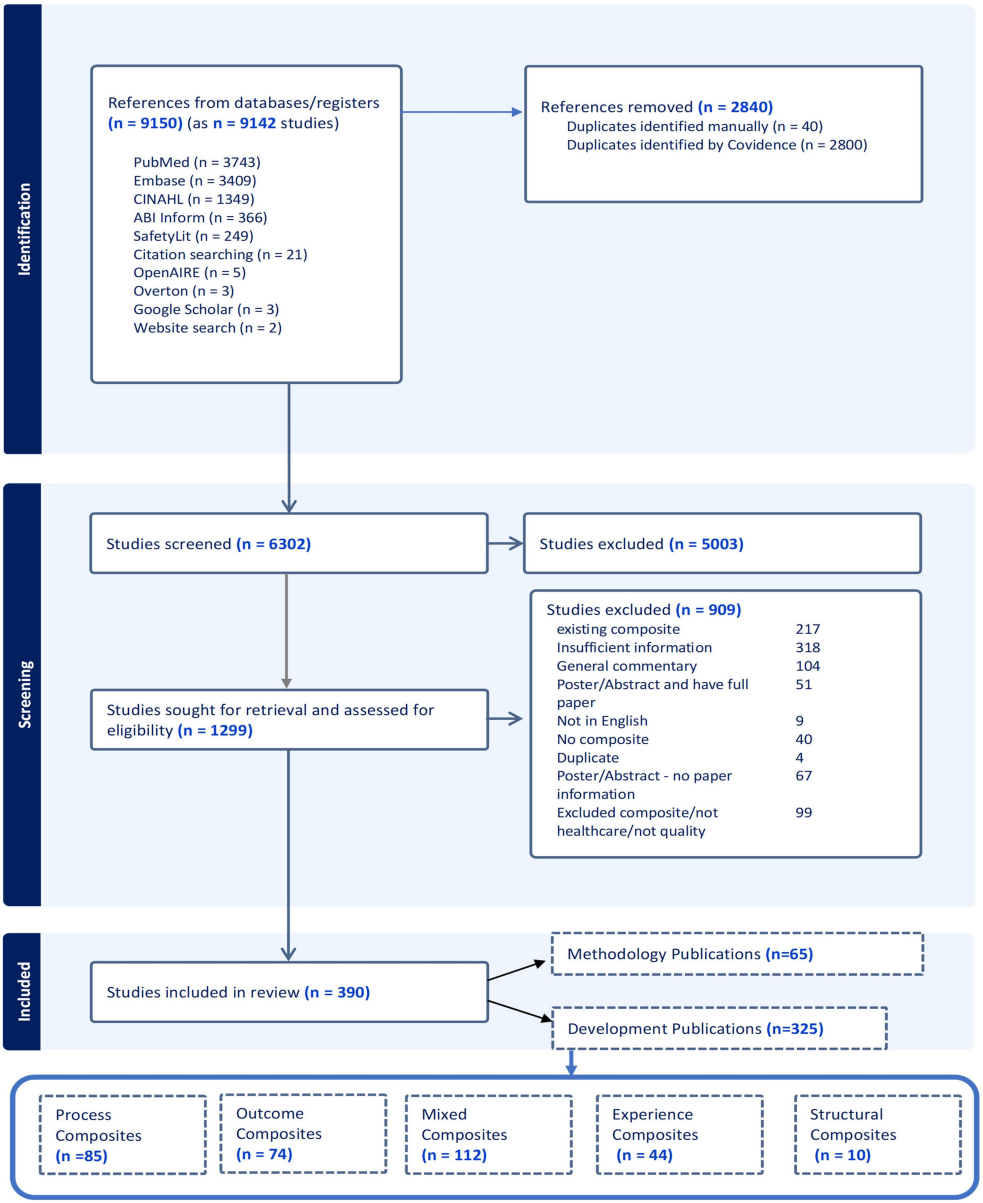
PRISMA diagram.

The final included studies for screening (Figure 1) were downloaded to EndNote 20 (n = 9,142) and duplicates removed (n = 2,840). Titles and abstracts of papers (n = 6,302) were independently screened by two reviewers using Covidence™. The initial screening was carried out by a team of 6 reviewers (TMcD, JKD, EMcA, CMcN, KMcD, MO’D), while 2 reviewers completed the updated screening. A screening guide detailing the eligibility criteria, including a decision tree, facilitated reviewers in making their assessment (Supplementary File 1). All potentially relevant articles (n = 1,299) selected by both reviewers were retrieved and the full text examined for eligibility by two reviewers. Documents sourced from grey literature searches without an abstract underwent full-text review. Disagreements between reviewers about inclusion at either title and abstract or full text review stage were resolved by discussion at regular review meetings.

### 2.3 Data extraction

Data extraction was performed by three authors (TMcD, JKD, AM). Data Extraction Templates were designed and tested in Covidence™. Data extracted on publications included author, year, country, study aim, study contribution, funding, and possible conflict of interests. Publications that offer a critical assessment and/or methodological guidance on any of the EC-JRC stages of development were grouped separately and underwent a more abridged data extraction. Additional information was extracted from publications that develop a composite measure: number, name and type of composites, included indicators, healthcare setting and speciality, role of composite in the paper, the Institute of Medicine (IOM) domains reflected in the composite, details of stakeholder engagement, and data on each of the 10 stages of development (Table 1) and a further stage, *post implementation review*, as specified in the protocol (15). Extraction templates were also designed in Elicit™ (19) to extract responses to predefined questions and statements for all papers that derived a composite measure. All data extracted manually or using Elicit™ was reviewed by the authors (TMcD, JKD), crossed checked to the publication and amended where needed.

**Table 1:** 11 developmental stages of a composite measure.

PHASE I: DEVELOPMENT		
1	Theoretical framework explained	Identifies a theoretical underpinning. Clearly defines what is being measured and provides the basis for both the selection and combination of variables into a meaningful composite indicator
2	Selection of constituent indicators	Selection should consider the importance, analytical soundness, measurability, feasibility and coverage of proposed individual measures
3	Preparation of data analysis	Check the underlying structure of the data (incl. outliers and directionality, where relevant)
4	Missing data	Missing data must be assessed, described, and addressed
5	Normalisation	The need for normalisation to render the constituent indicators comparable is assessed and, where appropriate, is carried out.
6	Weighting (statistical & participatory) & aggregation methods	Consideration is given to both weighting and aggregation when calculating the composite measures, reflecting the theoretical framework and the data properties. Consideration should be given to correlation between measures and the appropriateness of compensation.
PHASE II: ASSESSMENT & PRESENTATION		
7	Uncertainty analysis	Assess the robustness of the composite indicator to the effect of subjective choices and chance variation
8	Link to other metrics	Validation of the composite measure against other related measures
9	Deconstruction	Present the results for the individual indicators so as to reveal what is driving the composite indicator
10	Presentation & dissemination	Present the composite measure in a way that aids understanding. This should include use of visual tools such as tables and graphs, and the presentation of measures of uncertainty.
11	post-implementation review^a^	How the composite measure is reviewed once implemented to ensure continued relevance and effectiveness.

Adapted from Joint Research Centre-European Commission, Handbook on constructing composite indicators: methodology and user guide. 2008: OECD publishing (9).

a Not included in the information score.

### 2.4 Data synthesis

Publications that derive a composite measure of quality were categorised by whether the composite measure was derived from process measures only, outcome measures only, experienced/survey-based measures only, structural measures only, or a mix of all measure types (Table 2).

**Table 2:** Categorisation of composite measures by type.

TYPES OF COMPOSITE MEASURES	
Process composite	Consolidate process measures only, measures that indicate what a provider does to maintain or improve health, and these measures typically reflect generally accepted recommendations for clinical practice
Outcome composite	Consolidate outcome measures only, measures that reflect the impact of the health care service or intervention on the health status of patients
Structural composite	Consolidate structural measures only, measures related to a health care provider's capacity, systems, and processes to provide high-quality care (20)
Experience-based composite	Consolidate experience-based measures only, derived from surveys of patients, family members, staff or decision-makers, and represent a consolidation of responses to questions on either single of multiple domains, on topics such as communication, courtesy and respect, access to care, and care coordination (21)
Mixed composite	Consolidate measures of two or more of the above types

Given the broad nature of this scoping review, including many potential study designs and objectives, a critical quality appraisal was not performed. However, a scoring scheme was created to identify papers with significant information on a development stage. An information score (0, 1 or 2) for each of the 10 development stages was allocated, with 0 indicating the publication provides no relevant information on the stage, 2 indicating that comprehensive information on the stage is provided, while the intermediate score of 1 indicates the paper does provide information on this stage, but it is not comprehensive. The score is derived from the information presented in the publication, including clearly referenced connected papers from the same study. If the publication does not detail the required information to achieve a maximum score of 2 for that stage, it is assumed that these steps have not been taken. This score is not a reflection of the underlying quality of the paper, but rather the extent of information provided on the derivation of the composite measure.

A narrative synthesis is presented, supported by tables and graphs to summarise the findings, focusing on each of the 10 core developmental stages (Tables 3, 4), and commentary focuses on papers that achieve a maximum score (2) in a stage. An eleventh stage is also discussed, *post-implementation review*, to capture information on how composite indicators are reviewed once implemented to ensure continued relevance and effectiveness. This stage was not included in the information score as publications that included information on review were the exception.

**Table 3:** Examples of publications applying methods at each of the 10 stages.

Stage/Method	Type of Composite				
	Structural (n = 10)	Process (n = 85)	Outcome (n = 74)	Mixed (n = 112)	Experience (n = 44)
1. Theoretical Frameworks applied in the development of composite indicators					
*Maximum score (n)*	*3*	*None*	*7*	*28*	*11*
Developed from existing frameworks	(22, 23)		(24, 25, 44)	(10, 26–43, 45, 46)	(47–50)
Be-spoke frameworks	(51)		(52)	(53) (54–58)	
2. Selection of constituent indicators					
*Maximum score (n)*	*6*	*42*	*43*	*56*	*31*
Delphi or modified Delphi approach		(60–69)	(70, 71)	(27, 28, 37, 40, 59, 72, 73)	
Statistical analysis (e.g., factor analysis and PCA)	(74, 75)	(76–83)	(25, 84–91)	(31, 33, 41, 55–57, 92, 93)	(48–50, 94–118)
3. Preparation of data analysis					
*Maximum score (n)*	*9*	*75*	*67*	*84*	*42*
Factor analysis and PCA to group measures	(75)	(76, 77, 79, 82, 83, 119, 120)	(25, 70, 84–86, 90, 91, 121, 122)	(31, 33, 35, 36, 38, 41, 55–57, 59, 92, 93, 123–132)	(48–50, 95–97, 99–103, 106, 108–111, 113, 114, 117, 118, 133–136)
Hierarchical or mixed-effects regression	(51)	(137–141)	(70, 71, 87, 89, 142–154)	(29, 42, 155–161)	(105, 107, 112, 162–164)
Generalized estimating equations (GEE)		(165–172)	(173)	(174–177)	
Use of graphs to assess distribution and outliers (histograms, box-plots, funnel plots)		(60, 271)	(142, 180–186)	(187)	
4. Missing data: assessed, nature, method					
*Maximum score (n)*	*None*	*11*	*12*	*18*	*11*
multiple imputation		(68, 120, 166, 188, 189)	(85, 190, 191)	(29, 30, 177, 192, 193)	(49, 108)
Single imputation: mean/median/mode substitution		(172, 195, 196)		(72, 194, 197)	(100, 117)
Single imputation regression			(173)		
Single imputation: hot-deck imputation				(174)	(101, 198)
5. Normalisation: methods					
*Maximum score (n)*	*4*	*11*	*32*	*50*	*11*
Converted to categorical scales		(199, 200)	(71, 87, 121, 201–205)	(39, 42, 92, 158, 161, 206–210)	(48–50, 104)
Standardised	(211)	(138, 179, 195, 212, 213)	(85, 91, 122, 190, 191)	(10, 26, 43, 58, 72, 175, 194, 197, 214–217)	(117, 163)
Ranking		(218)	(52)	(129, 219–221)	
Min-max				(40, 174)	
Observed-minus-expected (O-E) score		(222)	(223)		
6. Weighting & Aggregation					
*Maximum score (n)*	*4*	*25*	*34*	*61*	*1*
Weighting: participatory methods	(23, 51, 74)	(60, 62, 69, 224–226)	(25, 154, 227, 228)	(40, 43, 54, 194, 207, 214, 215, 219–221, 229–234)	
Weighting: Statistical methods	(75)	(83, 120, 179)	(142, 191, 236)	(31, 41, 57, 125, 128, 130, 157, 237) (238–241) (242, 243) (123)	
Aggregation: additive^a^	(51, 74)	(172, 212, 244–246)	(228)	(53, 235)	
Aggregation: multiplicative^a^			(247)	(26, 72, 160, 248)	
Aggregation: non-compensatory^a^		(65, 172, 212, 244–246)	(181, 183–185, 249–252)		
7. Uncertainty Analysis					
*Maximum score (n)*	*3*	*11*	*17*	*22*	*11*
mechanism for including or excluding an indicator		(81)	(84, 91) (253, 254)	(36, 241, 255)	(48, 96) (99, 101–103, 107, 110, 111)
Data inclusion, structure and/or quality		(76, 81, 140, 170, 246, 256, 257)	(146–148, 258) (151, 173) (86, 143, 144, 153, 253, 259, 260) (254)	(123, 127, 261–263) (130, 214) (132, 216, 264) (39, 234)	(48, 99, 101–103, 107, 108, 110, 111, 114)
normalisation scheme	(211)	(213)	(84)	(39, 72, 160, 255, 265)	
imputation of missing data			(85, 173, 190, 266)	(43)	(94, 108, 111)
choice of weights	(23, 75, 211)	(213, 267) (120, 140, 268)	(146, 173) (85, 151, 253)	(53, 215, 234, 255, 262, 264, 269)	
aggregation method	(211)	(170, 213, 246, 257, 267, 268)	(84, 144)	(36, 39, 53, 72, 160, 175, 264, 265)	
8. Link to other metrics (validation)					
*Maximum score (n)*	*7*	*46*	*44*	*47*	*31*
Mortality or survival	(211)	(62, 65, 80, 120, 141, 165, 166, 168, 189, 213, 270, 271)	(25, 71, 148, 153, 181, 184, 185, 191, 203, 227, 251, 252, 260, 272–275)	(34, 54, 124, 132, 158, 206, 208, 217, 238, 239, 241, 261, 276–279)	
Morbidity		(65, 189)	(88, 153, 180, 204, 258, 260, 273, 274)		
Complications		(280, 281)	(89, 203, 251, 282, 283)	(206, 240)	
Hospital admissions or readmissions		(66, 81, 165, 170, 257, 268, 284, 285)	(25, 203, 223, 227, 273, 274)	(286)	
Hospital accreditation		(244, 245, 280)		(262)	
Experience scores		(76, 165, 199)	(287)	(158, 288)	
Global experience/ satisfaction measures					(48–50, 95–97, 101, 102, 107, 112, 114, 116–118, 162, 164)
9. Deconstruction					
*Maximum score (n)*	*6*	*45*	*46*	*64*	*32*
Correlation analysis	(23, 75)	(62, 64, 76–82, 119, 140, 141, 170, 179, 195, 212, 222, 226, 257, 268, 289–295)	(25, 71, 84, 85, 88, 90, 142–145, 148, 150, 153, 154, 184, 186, 203, 227, 247, 249–251, 253, 260, 266, 272, 287, 296, 297)	(29, 31–38, 45, 46, 53, 54, 56–59, 72, 92, 124–126, 128, 129, 131, 132, 157, 159, 161, 174–176, 187, 197, 206, 208, 216, 217, 230, 233, 234, 237, 240, 255, 261, 262, 264, 277, 278, 288, 298–303)	(47–50, 95–97, 100–102, 105–111, 113, 114, 117, 133–136, 162, 164, 198, 304, 305)
Dominance assessed	(23)	(63, 76, 78–82, 179, 212, 222, 306)	(25, 84, 88, 90, 142, 144, 148, 227, 249–251, 260, 266, 274, 283)	(37, 53, 57, 58, 72, 124, 129, 131, 157, 174, 175, 197, 208, 230, 234, 238, 262, 264, 299, 303)	(48–50, 97, 100–102, 107, 108, 111, 113, 114, 117, 135, 164, 305, 307)
10. Presentation and dissemination (format, metrics of uncertainty)					
*Maximum score (n)*	*4*	*38*	*48*	*65*	*6*
Bar and line graphs	(22)	(67, 120, 168, 294, 295, 308, 309)	(44, 88, 121, 145, 227, 249, 251, 252, 266, 275, 296, 310, 311)	(29, 30, 36, 92, 125, 126, 129, 130, 155, 157, 210, 231, 239–241, 248, 255, 261, 301, 312–314)	(98, 105, 116, 315)
Scatter plots		(76, 179, 218, 270)	(191, 203)	(26, 31, 38, 46, 57, 72, 123, 159, 176, 229, 302)	
Histograms		(60, 165, 170, 257, 337)	(151, 153, 154, 253, 259, 260)	(27, 28, 39, 41, 43, 53, 208, 219, 220, 229)	
Boxplots	(211, 317)	(289, 318, 319)	(84)	(187, 214, 278, 320)	
Caterpillar/Funnel Plot		(246, 306)	(144, 148, 150, 151, 247)	(174, 221, 234, 262, 264, 299)	
Forest plots		(140, 244, 290)	(153, 201)	(192, 321)	(163)

a A number of publications use one or more method of aggregation.

**Table 4:** Scoring criteria and publications with a Maximum information score (2) for any of the 10 stages.

Stage	Description	Instruction	Information Score Criteria	Publications with a Maximum Information Score (2)				
				Structure	Process	Outcome	Mixed	Experience
1	Theoretical framework	If the paper explicitly states that a theoretical framework is adopted, name and describe this framework. If not, state that no theoretical framework is explicitly stated. Explain the theoretical framework that provides the basis for the selection and combination of variables into a meaningful composite indicator under a fitness-for-purpose principle (involvement of experts and stakeholders is envisaged at this step). If stakeholders or experts are involved, explicitly state this. If not, state there is no mention of their involvement.	2: Explicit theoretical framework presented 1: Guidelines developed with expert/stakeholder involvement 0: No explicit framework of expert/stakeholder developed guidelines	(22, 23, 51)	None	(24, 25, 44, 52, 121, 122, 151)	(10, 26–43, 45, 46, 53–58, 72)	(47–50, 106, 109, 112, 133, 136, 198, 305)
2	Method of selection of constituent indicators	What methods are used to select indicators included in the composite(s) measures? Should be based on the analytical soundness, measurability, coverage, and relevance of the indicators to the phenomenon being measured and relationship to each other. The use of proxy variables should be considered when data are scarce. (involvement of experts and stakeholders is envisaged at this step).	2: Robust process of consideration and selection of metrics informed by expert opinion or based on statistical methods. 1: Robustly developed guidelines or similar used (incl CMS) relied upon (not developed by this study) 0: No information on indicator selection.	(22, 23, 51, 74, 75, 322)	(60–69, 76–83, 119, 137, 138, 140, 171, 172, 179, 188, 212, 213, 218, 222, 224–226, 267, 268, 280, 285, 294, 306, 308, 309, 324)	(24, 25, 44, 52, 70, 71, 84–91, 143, 146, 147, 150–154, 181–185, 191, 227, 228, 249–253, 259, 260, 273, 297, 310, 324–326)	(27, 28, 31–33, 35–37, 40, 41, 43, 45, 46, 53–59, 72, 73, 92, 93, 124, 125, 127–129, 131, 155, 157, 174, 187, 207, 209, 214, 219–221, 229, 231, 232, 234, 239–241, 243, 248, 261, 265, 300, 303, 313, 321, 327)	(48–50, 94–118, 136, 163, 164)
3	Preparation of data analysis	List the datasets used. Describe the data analysis conducted to assess the overall structure of the dataset to assess it’s suitability of the data to report the individual indicators accurately? Describe how subsequent methodological choices such as weighting and aggregation of indicators were informed by this analysis. Were outliers checked for? Was the directionality of indicators considered when combining measures in the composite?	2: Details of data analysis conducted to assess suitability of the dataset (e.g., EFA, need for clustering etc) and directionality or outliers considered if relevant (may not if using high quality data). 1: If structured of the data reviewed, but not in a comprehensive manner; 0: If data analysis not discussed or if analysis not relevant to indicators.	(22, 23, 51, 74, 75, 211, 322, 328, 329)	(60–63, 65–68, 76–83, 119, 120, 137–141, 165–172, 179, 188, 189, 195, 196, 199, 200, 212, 218, 222, 224–226, 244–246, 256, 257, 267, 268, 270, 271, 280, 284, 285, 289–295, 306, 318, 319, 330–338)	(25, 70, 71, 84–91, 121, 122, 142–154, 173, 181–186, 190, 191, 201–205, 223, 227, 236, 247, 249–254, 258–260, 266, 272–275, 282, 283, 296, 297, 311, 324, 325, 339)	(26, 29, 31, 33, 35, 36, 38–43, 45, 46, 53, 55–57, 59, 72, 92, 93, 123–132, 155–161, 174–177, 187, 192–194, 197, 207, 215–217, 220, 221, 232–234, 237–239, 240, 255, 261–263, 265, 269, 276–278, 286, 288, 299, 301–303, 312, 314, 320, 321, 340–343)	(47–50, 95–118, 133–136, 162–164, 304, 305, 307, 315, 344–346)
4	Missing data: assessed, nature, method	Is missing data assessed, described, and addressed using a method such as imputation?	2: If missingness is investigated and either described appropriately as nil/negligible or method used to address (e.g., imputation) 1: Missingness investigated and identified but not addressed. 0: Missingness not mentioned.	None	(61, 68, 120, 166, 172, 188, 189, 195, 196, 256, 289)	(71, 84, 85, 147, 148, 173, 181, 190, 191, 247, 266, 272)	(29, 30, 38–40, 43, 45, 46, 53, 72, 125, 174, 177, 192–194, 197, 340)	(49, 50, 94, 95, 100, 101, 103, 107, 108, 117, 198)
5	Normalisation methods	Detail the normalisation carried out to render the constituent indicators comparable by presenting them on the same scale (e.g., binary, standardisation). If the individual indicators are on comparable scales (e.g., means, z-scores, binary) this may not be required.	2 = Individual measures not on the same scale and standardisation applied 1 = On the same scale - no normalisation needed. 0 = No information	(23, 211, 322, 328)	(138, 179, 195, 199, 200, 212, 213, 218, 222, 225, 256)	(25, 44, 52, 71, 84, 85, 87, 91, 121, 122, 143, 146–148, 151–153, 186, 190, 191, 201–205, 223, 253, 259, 260, 297, 310, 326)	(26, 32, 33, 36, 37, 39–43, 45, 46, 53, 58, 72, 92, 123, 124, 129, 131, 156, 158, 161, 174, 175, 194, 197, 206–210, 214–217, 219–221, 229, 234, 237, 239, 243, 248, 262, 264, 265, 299, 340)	(48–50, 96, 104, 117, 133, 136, 163, 315, 344)
6	Weighting (statistical & participatory) & aggregation methods (for example additive, multiplicative, non-compensatory)	Detail the weighting and aggregation of individual indicators when calculating the composite measures. Detail if these weighting and aggregation procedure(s) respect both the theoretical framework and the data properties. Detail if the paper discusses whether correlation issues among indicators should be accounted for. Detail if the paper discusses whether compensability among indicators should be allowed.	2: Weighting detailed, aggregation clear, statistical analysis (exclusion/correlation) if relevant, or more than one approach (e.g., opportunity and all or none); 1: If simple proportion - no further information or discussion; Score 0 - If no information	(23, 51, 74, 75)	(60, 62, 65, 69, 76, 77, 79, 80, 82, 83, 119, 120, 172, 179, 195, 212, 224–226, 244–246, 256, 267, 291)	(25, 84–86, 88, 90, 91, 142, 143, 146–148, 152–154, 173, 183, 186, 191, 201, 203, 223, 227, 228, 236, 247, 253, 259, 260, 282, 297, 310, 311, 324)	(26, 31–33, 35–37, 40, 41, 43, 45, 46, 53, 54, 56, 57, 59, 72, 92, 123–132, 157, 160, 175, 194, 197, 207, 214, 215, 219–221, 229–231, 233, 234, 237, 240–243, 248, 261, 263–265, 269, 299, 340)	(133)
7	Uncertainty analysis	Should be undertaken to assess the robustness of the composite indicator in terms of e.g., the mechanism for including or excluding an indicator, the normalisation scheme, the imputation of missing data, the choice of weights, the aggregation method.	2 - Reasonable to comprehensive analysis conducted 1 - Some analysis conducted 0 - No uncertainty or sensitivity analysis	(23, 75, 211)	(76, 81, 120, 140, 170, 213, 246, 256, 257, 267, 268)	(84–86, 91, 143, 144, 146–148, 151, 153, 173, 253, 254, 258–260)	(36, 39, 53, 72, 123, 127, 130, 132, 160, 175, 214–216, 234, 241, 255, 261–265, 269)	(48, 96, 99, 101–103, 107, 108, 110, 111, 114)
8	Link to other metrics (validation)	Should be made to correlate the composite indicator (or its dimensions) with existing (simple or composite) indicators as well as to identify linkages through regressions.	2: Statistically linked to other related quality measures (e.g., regressions performed); 1: - Basic regressions/correlations but not against Q measures (e.g., assessment versus characteristics not specifically related to quality); 0: Nothing done to link	(22, 51, 74, 75, 211, 328, 329)	(61, 62, 65, 66, 68, 76, 77, 80–83, 120, 137, 141, 165, 166, 168, 170, 171, 188, 189, 195, 199, 200, 213, 224, 244–246, 257, 267, 268, 270, 271, 280, 281, 284, 285, 289, 291, 293, 330, 331, 334, 336, 348)	(25, 70, 71, 87–89, 121, 122, 142, 143, 145, 148, 152–154, 173, 181, 182, 184–186, 191, 203–205, 223, 227, 236, 247, 250–252, 258–260, 266, 272–275, 282, 283, 287, 297)	(29, 31–35, 41, 54, 123, 124, 126, 127, 131, 132, 156, 158, 159, 176, 187, 194, 206–208, 217, 220, 221, 238–241, 261–263, 269, 276–279, 286, 288, 298, 299, 301, 312, 320, 321, 340)	(47–50, 95–98, 101, 102, 104, 105, 107, 109, 112, 114, 116–118, 133, 134, 136, 162, 164, 198, 304, 305, 307, 344–346)
9	Deconstruction	Does the paper present the results for the individual indicators so as to reveal what is driving the composite indicator results? If yes, is this presented in a table, graph or in the text? Does the paper check for correlation between indicators and causality? Does the paper check if the composite indicator results are overly dominated by few indicators and to explain the relative importance of the sub-components of the composite indicator?	2: Clear presentation of constituent measures plus some determination of their role/relationship (correlation, dominance, relevance etc). 1: Presented but no meaningful discussion/analysis 0: n = No deconstruction	(22, 23, 74, 75, 211, 317)	(61–64, 68, 76–82, 119, 120, 140, 141, 170, 179, 189, 195, 199, 212, 218, 222, 226, 245, 246, 256, 257, 268, 270, 271, 289–295, 306, 318, 319, 348–350)	(25, 71, 84, 85, 88, 90, 121, 122, 142–145, 148, 150, 151, 153, 154, 183–186, 191, 201, 203, 205, 227, 247, 249–253, 259, 260, 266, 272–275, 282, 283, 287, 296, 297, 310, 311)	(29, 31–38, 41, 45, 46, 53, 54, 56–59, 72, 92, 124–126, 128, 129, 131, 132, 157, 159, 161, 174–176, 187, 194, 197, 206, 208, 214, 216, 217, 230, 233, 234, 237–239, 242, 255, 261, 262, 264, 276–278, 288, 298–303)	(47–50, 95–97, 100–102, 105–111, 113, 114, 117, 133–136, 162, 164, 198, 304, 305, 307, 344, 345)
10	Presentation & dissemination (format, metrics of uncertainty)	Does the paper present the composite measure results in a table that ranks the organisations or units? Are metrics of uncertainty presented e.g., standard deviation, confidence intervals? Does the paper present the composite measure results in a graph that ranks the organisations or units? What type of graph is used? Are metrics of uncertainty presented e.g., standard deviation, confidence intervals? Do the tables or graphs present trends in the composite measure(s) over time?	2: Table(s) and graph(s), plus measures of uncertainty. Tables or graphs that add to understanding of composite: ranking or trends. 1: Less comprehensive than 2 - table or graph (not both), possibly some measures of uncertainty. Score 0: No presentation of composite in table or graph.	(22, 23, 211, 317)	(60–63, 67, 76, 78, 83, 120, 139, 140, 165, 168, 170, 179, 196, 213, 218, 244–246, 256, 257, 270, 271, 289, 290, 293–295, 306, 308, 309, 318, 319, 331, 337, 338)	(44, 71, 84, 88, 89, 121, 144–149, 151, 152–154, 173, 181–186, 190, 191, 201, 203, 204, 223, 227, 228, 247, 249, 251–253, 259, 260, 266, 272, 274, 275, 296, 297, 310, 311, 324)	(26–31, 33, 36, 38–41, 43, 46, 53, 57, 59, 72, 92, 123–126, 129, 130, 155, 157, 159, 174, 176, 187, 192, 197, 208, 210, 214–216, 219–221, 229, 231, 234, 239–242, 248, 255, 261–264, 278, 299–302, 312–314, 320, 321, 352)	(98, 105, 116, 133, 163, 315)

Reporting was guided by the Preferred Reporting Items for Systematic Review and Meta-Analyses extension for scoping reviews (PRISMA-ScR) (353).

## 3 Results

Of the 6,302 studies screened, 1,299 were assessed for eligibility via full-text screening and 390 were included in the review. The most common reasons for exclusion were the publication used an existing composite (n = 217), insufficient information on composite development (n = 318), composite unrelated to quality or healthcare (n = 99), or the publication was a general commentary with no discussion of methods (n = 104). Of the included publications, 65 were methodological papers, with the remaining 325 publications developing composite measures of quality in healthcare. Of the publications developing composite measures, most studies were conducted in the United States (60%) and most within a hospital setting (57%).

### 3.1 Methodology publications

Methodology papers (n = 65) provide insight on the evolution of composite measures of quality, offer technical guidance on aspects of the development process, while also highlighting many of the key concerns raised about composite measures. Early discussions evolved from the introduction of hospital star ratings in the National Health Service (NHS) in the United Kingdom in 2001, highlighting the importance of understanding how the use of alternative methods can lead to significantly different rankings (354–357), with similar conclusions reached on rating general practitioners (316). While star ratings were seen by some management as useful to drive beneficial change, the ratings were also reported to have sometimes induced unintended and dysfunctional consequences (358).

In the United States, methodologies adopted by the Centers for Medicare & Medicaid Services (CMS) have been scrutinised and challenged for the past 20 years. Concerns were raised about poor discrimination, lack of measure balance, and lack of correlation with important clinical outcomes (359), and inconsistency between constituent measures and the composite (360). However, focus has primarily been on weighting, aggregation and normalisation choices, placing hospitals within appropriate peer groups, with many publications exploring the impact of using alternative methods (361–374). The review by the CMS of its hospital ratings sparked criticism of vagueness, issues with included measures, bias against hospitals that serve socio-economically disadvantaged areas, and how reporting fewer measures can result in higher ratings (375–379). The problem of excluding measures for low-volume hospitals is discussed in terms of implications and methodologies to more accurately present ratings (351, 380), while alternatives to the latent variable model (LVM) used by the CMS are also discussed (381, 382). Following significant public consultation (378), CMS announced in 2019 that it was removing the LVM statistical model, opting to simplify and improve transparency, reducing the number of measure groups, standardising the measure group and placing hospitals into peer groups characterized by the number of measures submitted (383). However, once the CMS technical specifications were altered and recalculated using reasonable alternative methods, on average, half of hospitals were assigned a different star rating, illustrating how sensitive results are to methodological choices and methods applied, raising questions about their design and transparency (5, 384).

Elsewhere, Couralet et al. determine the impact on French hospital ranking of difference aggregation methods. Moore et al. compare three composite methods that are widely used in other health care domains to identify the most appropriate for trauma care process performance evaluation in Canada (385, 386). Using Dutch data, van Doorn-Klomberg et al. find that a composite performance score has increased precision over individual indicators, and a small number of indicators can be sufficient to achieve this level of precision (387).

Finally, a number of publications provide methodological guidance across a number of stages of development of composites (3, 4, 178, 388–393), others raise particular concerns about composites (6, 8, 394, 395), and others consider specific stages for particular types of measures (392, 396–409). Many of these publications are further discussed in the coming analysis.

### 3.2 Publications developing composite measures

**Figure 2:**
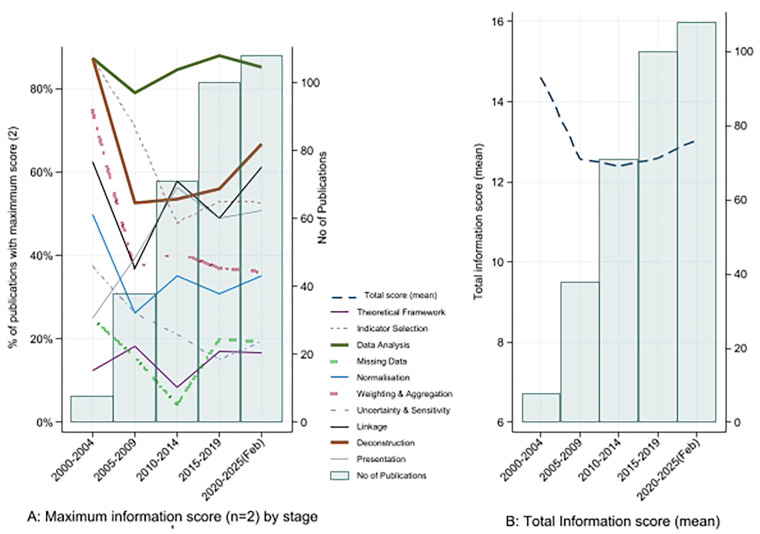
Trend in information scores and number of publications (2000-2025): (Panel **A**) Percentage of papers with a maximum score for each stage (trend lines) and number of publications (bars); (Panel **B**) mean information score (trend line) and number of publications (bars).

The number of publications has risen steadily over the 25 years of this review. Figure 2 illustrates the evolution of publications and maximum information scores by stage from January 2000 to February 2025, summarised over blocks of 5 years. Early publications (2000-2004), though few in number, generally score better than publications between 2005 and 2014, however scores across most categories improved in the period 2015–2025. This coincides with rapid advancements in information technology and data availability, which facilitate an increased focus on the measurement of quality. Supplementary Files 2, 3 provide a full list of included publications and details of country, healthcare setting and year of publication.

**Figure 3:**
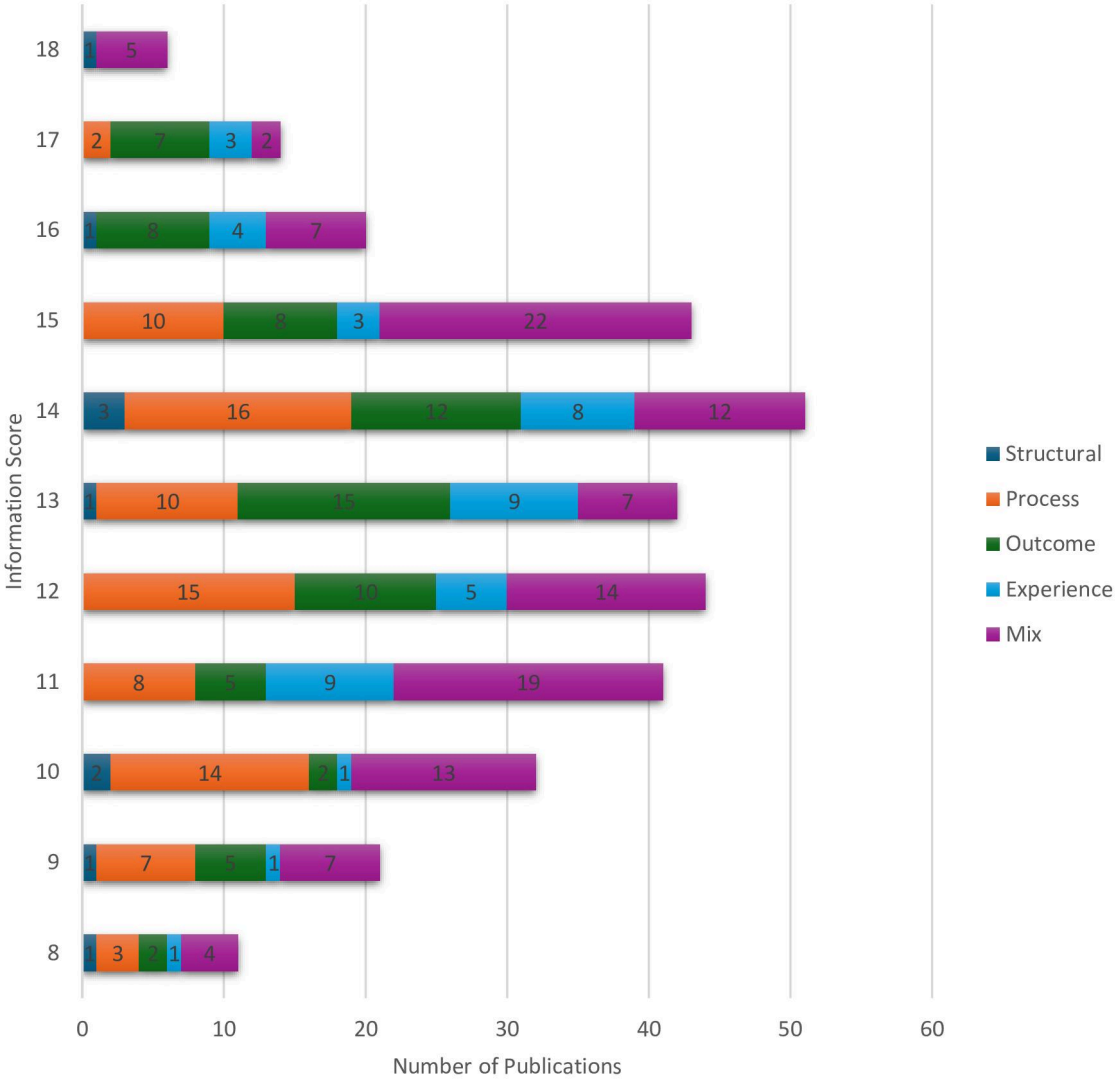
Distribution of total information score.

Publications that derive a composite measure of quality were categorised by whether the composite measure was derived from process (n = 85), outcome (n = 74), experienced/survey-based (n = 44), structural (n = 10), or a mix of process, outcome, experience or structural measures (n = 112). The information score is calculated for all 10 stages for each publication that develop composite measures (Figure 3).

While the overall median score is 13, papers developing process and mixed composites have a lower median (12), compared to those developing experience (13), outcome (13) and structural (13.5 measures). Supplementary File 4 details scores for all papers across all 10 stages. Most publications do perform some level data analysis (median 2, mean 1.85), publications often score well on measure selection (median 2, mean 1.54), deconstruction (median 2, mean 1.53) and presentation (median 1, mean 1.46). Scores for framework (median 1, mean 0.86), missing data (median 1, mean 0.71) and uncertainty analysis (median 1, mean 0.74) are generally low. The extent and nature of information reported under each of these stages for included publications is analysed.

#### 3.2.1 Identify the theoretical frameworks applied in the development of composite indicators

The theoretical framework provides the scaffolding for developing a composite measure. A framework should clearly define what is being measured and provide the basis for both the selection and combination of variables into a meaningful composite indicator. The median score for this stage is 1 (mean 0.86), with 49 publications assigned a maximum information score. Many papers mention Donabedian's structure, process and outcome indicator classification (410), and/or the Institute of Medicine's (IOM) framework six domains: safety, effectiveness, patient-centredness, timeliness, efficiency and equity (2), however few composites are derived using these frameworks.

As shown by Table 3, a modest number of papers construct a framework to underpin the development of a mixed composite (53–58), it is more common for existing frameworks to be considered and moulded to guide measure development (10, 26–43, 45, 46). Profit et al. draw on existing frameworks, including the IOM and Donabedian classifications, to model the patients path through the health system, and propose a quality matrix to support the identification of structural, process, and outcome metrics for five of the six IOM domains of quality (10). The matrix is presented within a structure that identifies health determinants, health system factors and illness severity as moderators that must be considered when developing the composite. Austin et al. detail how the conceptual framework underpinning Leapfrog's composite patient safety score for U.S. hospitals was developed, outlining discussions on what the composite should measure, the nature of included measures, the weighting to be applied and the selection process (43). Shahian et al. develop a conceptual framework for selecting and categorizing quality measures, presenting principles for measure selection, consideration of structure, process and outcome measures, identifying domains of care, and detailing measures considered but not included (53). This framework forms the basis of the development of related measures by the Society of Thoracic Surgeons in the US and Europe (45, 46).

Frameworks for the development of composite outcome measures have been developed from strategy documents (24), existing frameworks (25, 44), and be-spoke conceptual frameworks (52), while structural composite measures are derived from literature review and experts’ discussion (51), and existing frameworks such as the WHO health systems ‘building blocks’ (WHO BB) (22) and the WHO determinants of accessibility (23). Theoretical frameworks for experience-based composites measures tend to be grounded in formative qualitative research, using interviews and focus groups to identify domains and constituent measures (47–50).

The lack of coherent theoretical models for composite measures is often critiqued (8, 394). However, guidance is available. Schӧner et al. adopt the OECD development approach as a general toolkit for technical and methodological issues, with the framework of Shwartz et al. providing relevant considerations in a healthcare context (389, 393). The National Quality Forum (NQF) advises whether “composite development begins with a conceptual construct of quality or with a set of measures one wishes to combine, the selection of the component measures should be conceptually coherent” (4).

#### 3.2.2 Methods of selection of constituent indicators for inclusion in composite indicators

This is one of the most important steps in developing a composite measure (391). Constituent measures of composite measures used for assessment of performance are often held to a high standard, for example the requirements set by the NQF (411). The selection of metrics may be informed by expert opinion or based on statistical methods (10). Examples publications are referenced by Table 3.

Papers that detail a robust process of consideration and selection of metrics informed by expert opinion or based on statistical methods are attributed a maximum information score within this stage (n = 178). The median score for papers deriving all types of composites is 2 (mean=1.54). While the identification of measures for consideration often involves review of literature and existing guidelines, expert opinion is also frequently garnered, while statistical methods such as FA and PCA are used to assess the relative relevance of constituent measures.

Methodological papers identify issues that should be considered when developing a composite measure. If the purpose of the measure is to compare performance, all measures used must be equally applicable across units being measured (8). The intended audience and purpose of the composite performance measure should be explicitly stated. This will provide the basis for selection of individual indicators and provide direction for methods of aggregation and weighting (388). Component measures may need to be adjusted for case-mix (388). Measures with a relatively high proportion of signal variance are useful because of their high power for discriminating among providers (359, 388). Where units do not return a measure this is problematic in terms of interpreting results (379, 401), with composite measures potentially misrepresenting quality across units and introducing bias (380). Chung et al. illustrate the use of hierarchical cluster analysis on denominator counts to group by number and types of measures, in effect comparing hospitals that take the same (or similar) test (370).

#### 3.2.3 Preparation of data analysis

The nature of the underlying data needs to be understood before the construction of a composite indicator, as the accuracy and credibility of the measure is dependent on data quality (9). Analysis may include checking for outliers and consideration of the directionality of indicators. Sample size may also be a concern as statistical analysis requires sufficient data (9). A maximum information score (Table 4) is assigned to publications that present a clear analysis of the structure of the underlying data (median=2; mean=1.85), with a significant number of papers achieving a maximum score (n = 277).

The extent of analysis needed will vary with the complexity and quality of the underlying data. The analysis should inform decisions on normalisation, weighting and aggregation. An exploratory analysis may include assessing the association between variables, the need for clustering, the consistency of the direction of indicators, and the existence and impact of outliers (9). Data analysis often involves summarizing measures using frequencies, percentages, and confidence intervals distribution, by calculating mean, median, variance, and interquartile ranges. Descriptive statistics are generally presented to assess the overall structure of the dataset. Sub-group variation is assessed using ANOVA, Pearson chi-square and Fisher's exact tests.

FA and PCA are used to assess data structure and group quality measures into categories, (Table 3), with metrics such as Cronbach's alpha assessing reliability. Regression models, including random and fixed-effect models, allow adjustment for case-mix and are used by to understand the structure of the data, identify relationships and assess variation (Table 3). Hierarchical or mixed-effects regression is often used to account for variability across units, while generalized estimating equations (GEE) is also used to account for within-site correlation and clustering (Table 3). Histograms and box-plots graphs are used to assess normality and variability of constituent indicators, and funnel plots are also used to identify outliers (Table 3).

#### 3.2.4 Missing data: assessed, nature, method

Composite measures developed from datasets with missing data may provide inaccurate and biased results if the nature and extent of missingness is not investigated, understood and, where appropriate, addressed.

A maximum information score under this category (Table 4) is achieved by a modest number of papers (n = 52), with multiple imputation the most often cited method (Table 3). The median score for this stage is 1 (mean 0.71), indicating that most papers do not mention consideration of missing data. While missingness may not be an issue if a composite measure is developed from a high-quality dataset, for example a national disease-specific registry with robust data quality processes (60), lack of comment may also mean that the extent and potential impact of missing data has not been considered. Single imputation is applied through mean/median/mode substitution, regression and hot-deck imputation (Table 3). Methods such as use of a proxy variable (266), exclusion from analyses (247, 272, 289), and assuming missingness indicated a process was not completed or an outcome was not achieved (61, 174) were also applied. Milliren et al. replaced zero rates with the negative log of the maximum across all hospitals for a given measure (84). Sensitivity analysis was occasionally carried out to assess the impact or choice of imputation method adopted (43, 94, 190, 266).

#### 3.2.5 Normalisation: methods

Normalisation will be required prior to any data aggregation if the indicators in a data set have different measurement units. Standardisation (or z-scores), ranking, min-max, distance to a reference measure and conversion to a categorical scale are all methods adopted (Table 3). The choice of method should be informed by the distribution of the data, such as the presence of outliers and skewness.

The median score for this stage for all types of composite measures is 1, with a maximum information score under this category (Table 3) achieved by a limited number of publications. Process and outcome indicators are generally derived from individual patient-level binary indicators either aggregated at patient or unit (e.g., hospital) level, for example percentage of patients who are on aspirin given within 24 h of arrival (412), or optimal pancreatic surgery defined as the absence of postoperative-mortality, serious morbidity, and a number of defined adverse outcomes (296), and normalisation is not required. However, a limited number of measures have been normalised before aggregation.

Shwartz et al. describe both z-scores and range scores as useful approaches to rescaling measures, and while five-star categorizations are easily communicated and reduce users’ cognitive burden, they can be unfair to facilities whose scores are near the category thresholds (389). However, the application of threshold-based classification rules to standardise disparate individual measures to categorical may reduce statistical power and potentially hide important differences and, as a “step forward” advises that measures should be standardised to consistent scales in a principled way that preserves the useful information in the underlying measures (8, 413).

#### 3.2.6 Weighting and aggregation methods

The attribution of weights reflects the relative importance of constituent measures, while the choice of aggregation focuses on whether poor performance in one metric may be compensated by superior performance in another (10). Publications that present clear details of both weighting and aggregation, statistical analysis (exclusion/correlation) if relevant, and/or consider more than one approach are assigned a maximum information score (n = 125).

Weights may be determined by participatory or statistical methods (Stage 2). Statistical methods include PCA and FA, however these methods are often used to assess dimensionality and validity but less frequently used to derive weights (Table 3). Regression-based weights are sometimes applied using Bayesian regression modelling, drawing the weighing from the underlying data (142, 191, 238–241, 388). Data envelopment analysis (DEA) uses mathematical programming to measure and evaluate the relative performance of several units based on an ‘efficiency’ score (236, 242, 243, 264, 414). Benefit of the doubt (BOD), an application of DEA, assigns higher weights to indicators on which performance is better (197, 363, 385). Other statistical methods used to derive weights include item response theory (178, 179, 235, 361) and Grey Relational Analysis (GRA) (123).

There are three principal choices when aggregating indicators: additive, multiplicative and non-compensatory, with the decision focusing on whether providers should be allowed to compensate for poor performance in one metric with superior performance in another (Table 3).

Composite measures may be a product of more than one method of weighting and aggregation. Shahian et al. and O’Brien et al. initially apply all-or-none to individual outcome and process-specific domains before rescaling and using additive aggregation to derive the final mixed composite (53, 235). Shahian et al. utilise a combination of aggregation methods to achieve the final composite (143).

Ranks were computed using 5 different weighting and aggregation schemes by Profit et al. (264). Equal weights and additive aggregation were the base case, with mean and median weights derived by experts and geometric (multiplicative) aggregation explored. Papers that compare different methods generally find that results varied considerably depending on the method of aggregation used (212, 264, 316, 362, 385, 386). Milliren et al. uses multiple methods for weighting and aggregation, including individual measure rankings, median and geometric mean rankings, and PCA-based summary scores (84). Shwartz et al. discuss a number of weighting and aggregation methodologies (144, 360, 363–366, 389) and provide a summary of the main advantages of each approach (389). Equal weighting is the most intuitive; opportunity-based weights create an equal incentive to focus on each measure and adjust for case-mix differences; numerator-based weights create a strong incentive to focus on the most prevalent measures; expert panels can make judgements about dimensions such as the level of harm associated with each measure; and all-or-none measures are patient focused and the most consistent with a commitment to the highest quality.

#### 3.2.7 Uncertainty and sensitivity analysis

Uncertainty analysis assessed how uncertainty in the input factors affects the composite indicator values, while sensitivity analysis assesses the contribution of the individual source of uncertainty to the output variance (9). Table 3 presents examples of the assessment of uncertainty due to decisions made about measure and data selection, data editing, data normalisation, weighting scheme, weights’ values and aggregation and the treatment of missing data. Uncertainty can arise also arise due to statistical noise, therefore composite indicators should be presented with accompanying displays of statistical uncertainty (8).

A maximum information score is assigned to publications that conduct a reasonably comprehensive analysis or explicit analysis (n = 64). The mean score for this stage is the second lowest of all the 10 stages across all classifications of composite measures 0.74 (median=1). Most publications that conduct an uncertainty or sensitivity analysis assess the impact of weighting and aggregation decisions (Table 3).

Wilhelm et al. created alternative composite indices that varied in one decision step from the base index, assessing the robustness of the composite score by evaluating the impact of different methodological choices, such as normalisation and aggregation methods, on facility rankings (72). Oluwatola e*t al.* created alternative scenarios to assess robustness at three stages: initial data analysis, normalisation, and aggregation. Variables were treated ordinally instead of dichotomously to assess impact, z-score standardisation was used as an alternative normalisation method, and geometric aggregation was used as an alternative to additive aggregation (39).

#### 3.2.8 Link to other metrics (validation)

Linking the composite measure to other external measures can test the explanatory power of the measure (9). Validity testing includes determining if the measure adequately distinguishes between good or poor quality as assessed by another valid method, and this can be ascertained by correlation analysis or by determining the ability of the measure to predict scores on some other related valid measure (4). For example, if the goal of the composite performance measure is to identify hospitals with excellent outcomes, then criterion validity might be assessed by comparing predictions based on the composite performance measure with observed outcomes, a gold standard or external criterion, if available (388).

A maximum information score is assigned to publications assessing a composite's link to other related quality measures (n = 175). The median score for this stage is 1 (mean=1.37). Table 3 details examples of measures of quality used to assess the validity of composite measures.

#### 3.2.9 Deconstruction

Composite measures should be deconstructed such that the contribution of subcomponents and individual indicators can be identified (9). Correlation analysis ensures the relationship between constituent measures and the composite is understood (Table 3). It is also important to assess if the composite indicator results are overly dominated by one or more indicators (Table 3).

A maximum information score is assigned to papers that clearly present individual results for constituent measures, assess correlation and dominance (n = 193; mean=1.63; median=2). Papers that use FA or PCA tend to present a clear deconstruction, with loadings indicating the relative influence of individual measures. While there is no optimal way to present individual indicators, deconstruction can be presented effectively using graphs (9), with radar or spider charts used effectively in a small number of papers (36, 133, 145, 214, 242, 276).

#### 3.2.10 Presentation and dissemination (format, metrics of uncertainty)

The format and extent of data presented on a composite measure generally depends on the nature of the composite and the purpose of the publication, with those focused on the development of a composite generally presenting more information (Table 3). Tabular format is often the most straightforward way in which composite measures are presented, with metrics of uncertainty (e.g., confidence intervals, standard error, statistical significance) and/or variability (e.g., mean, median, standard deviation, interquartile range) often included. Publications developing measures for cross-unit comparison may present information on ranking, with occasional presentation of temporal trends in composites.

A maximum information score is assigned to papers that present details of the composite in both tabular and graphical form (n = 161; mean=1.46; median=1). The tables and graphs must add to the understanding of the composite and may present rankings or trends over time. While bar and line graphs are widely used, many other types of graphs are used to present composite measures (Table 3).

#### 3.2.11 Post implementation review

Periodic review of all individual and composite measures should also be conducted to ensure measures remain valid and relevant, and to ensure decisions are made to revise or withdraw where necessary (8, 384). However, while few composites undergo a formal review and update (53, 58, 235, 273, 347, 415), some publications do outline future validation work (45, 95, 213, 298, 307), the expansion of the composite to include additional measures (146, 213, 229), propose a pathway for future development (201), and propose regular review (43, 46, 85, 297, 310, 398).

### 3.3 Addressing the gaps

Methodology papers offered technical guidance on the development process, while also highlighting concerns raised about composite measures. These papers, together with the framework provided by the OECD Handbook on Composite Indicators, provided a backdrop against which included publications were assessed across each of the 11 stages of development of composite measures (9). While no paper achieved a maximum score across all categories, 6 papers scored a maximum score across 8 categories (23, 41, 46, 53, 72, 124). High scoring papers clearly address and describe measure selection, data analysis, normalisation, weighting and aggregation, deconstruction, and presentation. However, the treatment of missing data and uncertainty analysis are the stages least likely to be well described by high scoring papers, and also the most likely to score zero for low scoring publications.

**Figure 4:**
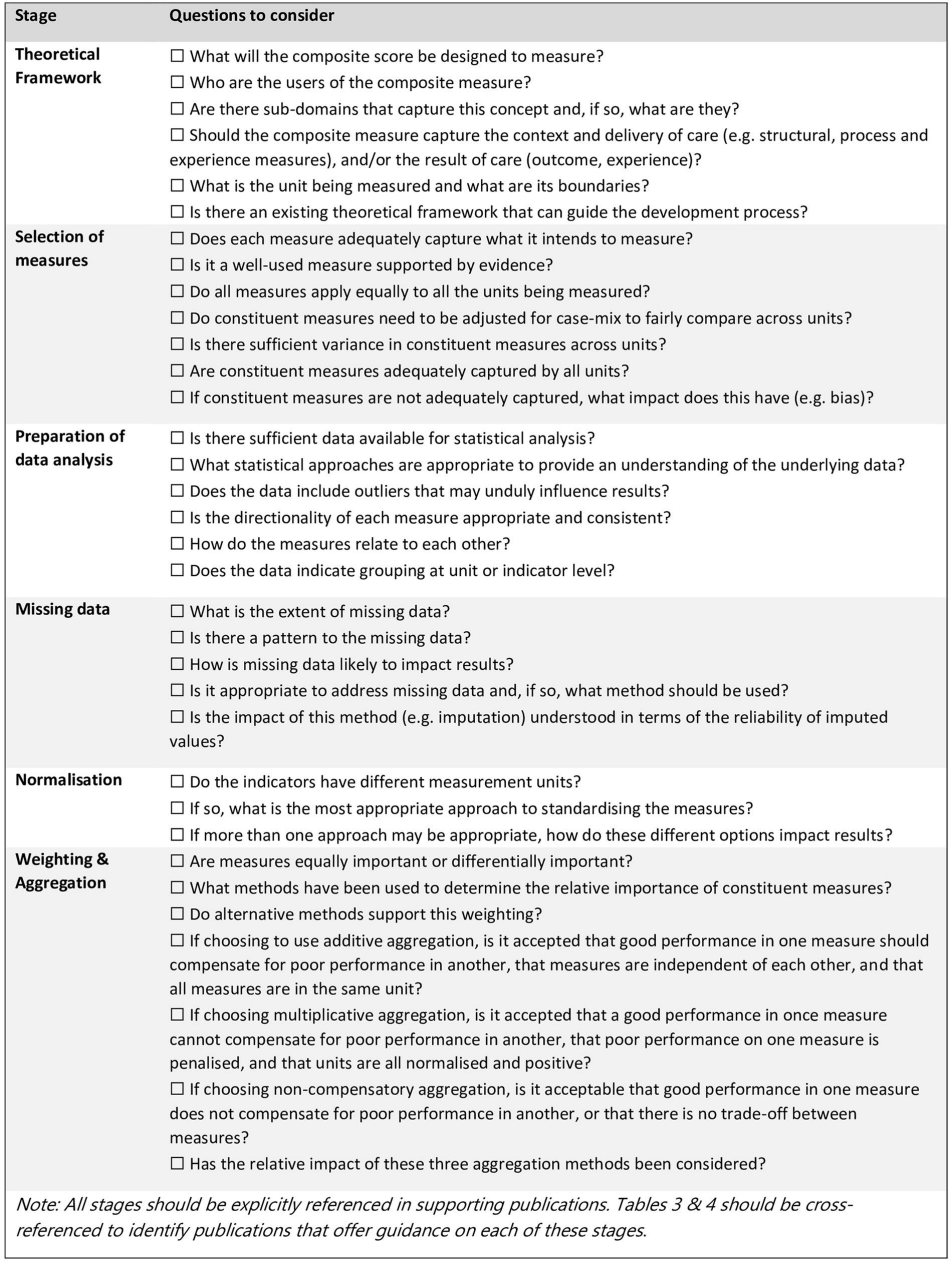
Phase I checklist – composite measure development.

**Figure 5:**
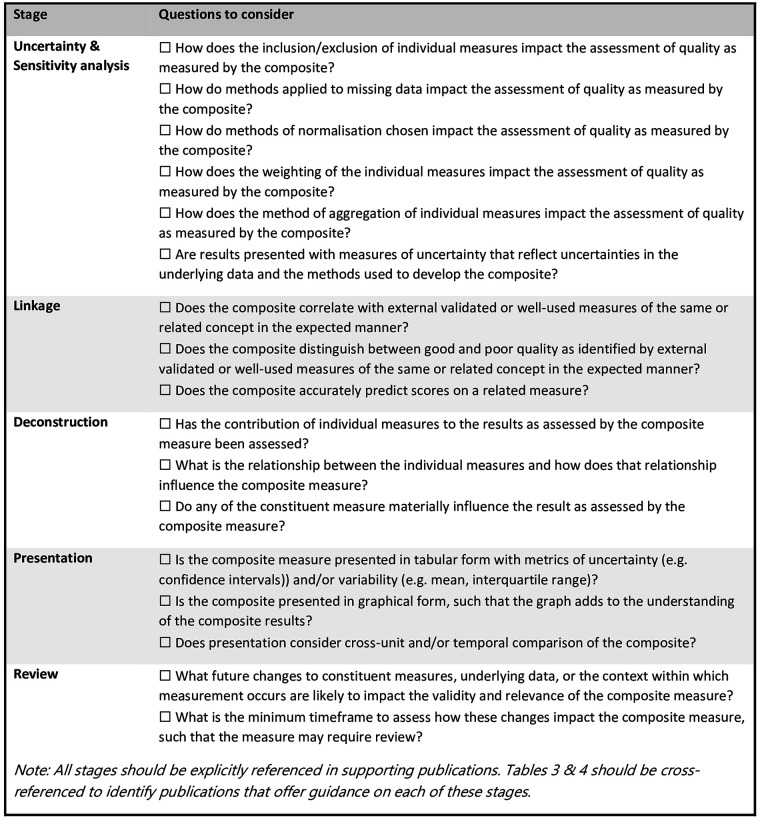
Phase II checklist – composite measure assessment, presentation & review.

This review captured the methodologies adopted and the methods applied to the derivation of composite indicators in this wide-ranging literature, identifying many examples of best practice across each of the stages of development. The key considerations under each stage were identified from the synthesis of the most informative publications (information score=2) and are presented as checklists to guide development across two distinct phases:

- Phase I (stages 1-6) (Figure 4) - composite measure development
- Phase II (stages 7-11) (Figure 5) - composite measure assessment, presentation and review.

These checklists provide an actionable framework to ensure composite measures of quality in healthcare are developed in a robust and transparent manner. The many examples signposted by Table 4, with specific methods identified by Table 3, provide both developers and users of composite measures with the guidance necessary to respond to the questions raised in these checklists.

## 4 Discussion

The use of composite measures of quality in healthcare is widespread. While their origin derives from the measurement of comparative performance at a national level, their use has expanded to the measurement of quality in many contexts and specialisms over the past 20 years. However, significant concerns have been raised about composite measures. The Health Foundation in England conducted an independent review of approaches to measuring the quality of general practice, and concluded that the development and dissemination of composite scores should be discouraged (7). This review raises many concerns including the heterogeneity of users, the level of subjectivity in the development process, the quality and appropriateness of available data, and the inability of composite measures to signal changes in care quality that are specific enough to be the target of improvement projects. While these comments relate to general practice, they may be applicable to composite measures used in many other healthcare contexts. The Foundation's review suggests a better approach to grouping data might be to allow users to self-select groupings from a menu of indicators. Presentation in dashboard formats could also aid transparency and allow the significance of measures to be understood collectively and individually (416). The use of data-driven signalling, facilitated by recent developments in artificial intelligence, could support the clustering of measures which collectively trigger a concern not identifiable from either the individual measures or a single composite.

However, composite measures are appealing as they can help condense this vast amount of information into a single indicator, which is easy to use and promises an overview of performance (4). Greater availability of data and an increased focus on quality measurement have resulted in a proliferation of quality measures, which can result in information overload. Composite measures summarising a range of quality dimensions may be helpful for patients, who tend to place greater importance on several different aspects of quality, namely they want care that is effective, safe, patient centred and delivered compassionately (6). Efforts to investigate key issues related to the construction of composite measures often rely on the opinions of experts who tend to focus on technical issues of composite construction (i.e., the statistical properties of the measures) (4, 388). The opinions of “on-the-ground” practitioners gathered by Martsolf et al. suggest that to ensure composite measure acceptance and use, stakeholders supported approaches should prioritise simplicity over methodological complexity, promote transparency, and follow a stringent “vetting” process (395).

Designing composite quality and safety measures involves many technical decisions. Through the adaption of the 10-stage methodology developed by the EC-JRC, combined with the application of an information score to select informative publications, this scoping review identifies the methods applied to the derivation of composite indicators used for the measurement and monitoring of quality and safety in healthcare. The actionable checklists, when used with the signposting provided by this scoping review, act as a practical toolbox for those developing, assessing and using composite measures. However, this review does have limitations. The decision to conduct and present a comprehensive survey of the literature both in terms of the number of publications that are included and the extent of data extracted precludes an in-depth consideration of some of the more technical challenges. Restricting included publications to those published in English is a further limitation. However, as approximately 1% of papers identified at title and abstract review stage were published in languages other than English, the omission of these papers is unlikely to materially alter the reported findings. Furthermore, as the information score identified publications that present high quality information on at least one of the stages, many publications did not meet our inclusion criteria. Nonetheless, the extensive signposting offered throughout this review does allow for greater exploration of these more challenging considerations where needed. Future research should focus on detailed guidelines about the development, design and reporting of composite measures.

## 5 Conclusion

While the use of composite measures can be controversial due to concerns around transparency, appropriateness and uncertainty, once the appropriateness of a composite measures has been established by stakeholders, this scoping review provide an actionable framework to aid the development of composite measures of quality in healthcare in a robust and transparent manner. The identification of many examples of best practice drawn from this wide-ranging literature will support the use of actionable checklists, greatly assisting those developing and interpreting composite measures.

## Data Availability

The original contributions presented in the study are included in the article/Supplementary Material, further inquiries can be directed to the corresponding author.
