# Supplementary material for: Methods to derive composite indicators used for quality and safety measurement and monitoring in healthcare: a scoping review
Source: Front Health Serv. 2026 Aug 4;6:1852718. doi: 10.3389/frhs.2026.1852718 (PMC13481381; doi:10.3389/frhs.2026.1852718)

Supplementary File 1

Search Criteria and Decision Tree

**SEARCH STRATEGY FOR ALL DATABASES: SEARCH DATE 16TH JANUARY 2023;**<br>***NOTE: SECOND SEARCH CARRIED OUT ON 10TH NOVEMBER 2025 COVERED THE PERIOD 1/1/23 TO 28 FEBRUARY 2025.***	
PUBMED	(("composite indicator*"[tw] OR "composite score*"[tw] OR "composite measure*"[tw] OR "composite index*"[tw] OR "star rating*"[tw] OR "single metric" [tw])<br><br>AND ("safety"[tw] OR "quality"[tw] OR Quality of Health Care[MeSH] OR Patient Safety [MeSH])<br><br>AND ("health care"[tw] OR "healthcare" [tw] OR "health-care" [tw] OR "Nursing Home*"[tw] OR "Hospital*"[tw] OR "General Practice"[tw] OR "primary care*" [tw] OR "family practice"[tw] OR "clinic" [tw] OR "clinics" [tw] OR General Practice[MeSH] OR Family Practice[MeSH] OR Primary Health Care [MeSH] OR Hospital [MeSH] OR Nursing Homes [MeSH]) AND ("2000/01/01"[Date - Publication] : "2022/12/31"[Date - Publication]))
CINAHL	((“composite indicator*” or “composite score*” or “composite measure*” or “composite index*” or “star rating*” or “single metric*”)<br><br>AND (quality or safety or MH “Quality of Health Care+” or MH “Patient Safety+”)<br>AND (“health care” or healthcare or “health service*” or hospital* or “medical care” or “general practice” or “primary care” or “family practice” or “nursing home*” or clinic or clinics or MH “Family Practice+” or MH “Health Service+” or MH “Health Facilities+”) AND LA english AND DT 2000-2022
EMBASE	('composite measure*':ti,ab,kw OR 'composite indicator*':ti,ab,kw OR 'composite score'/exp OR 'composite score*':ti,ab,kw OR 'composite index*':ti,ab,kw OR 'star rating*':ti,ab,kw OR 'single metric*':ti,ab,kw)<br><br>AND ('quality':ti,ab,kw OR 'safety':ti,ab,kw OR 'health care quality'/exp OR 'patient safety'/exp)<br><br>AND ('health care':ti,ab,kw OR 'primary care':ti,ab,kw OR 'nursing home':ti,ab,kw OR 'general practice':ti,ab,kw OR 'hospital*':ti,ab,kw OR 'family practice':ti,ab,kw OR clinic:ti,ab,kw OR clinics:ti,ab,kw OR 'hospital'/exp OR 'general practice'/exp OR 'primary health care'/exp OR 'nursing home'/exp) AND [english]/lim AND [2000-2022]/py
ABI/INFORM<br>(through Proquest)	noft("composite measure*" OR "composite indicator*" OR "composite score*" OR "composite index*" OR "star rating*" OR "single metric*") AND noft(quality OR safety) AND noft("health care" OR healthcare OR hospital* OR "general pract*" OR "primary care" OR "family pract*" OR "nursing home*" OR clinic OR clinics)
SafetyLit	(composite indicator* OR composite score* OR composite measure* OR composite index* OR star rating* OR single metric) AND (safety OR quality)

DECISION TREE

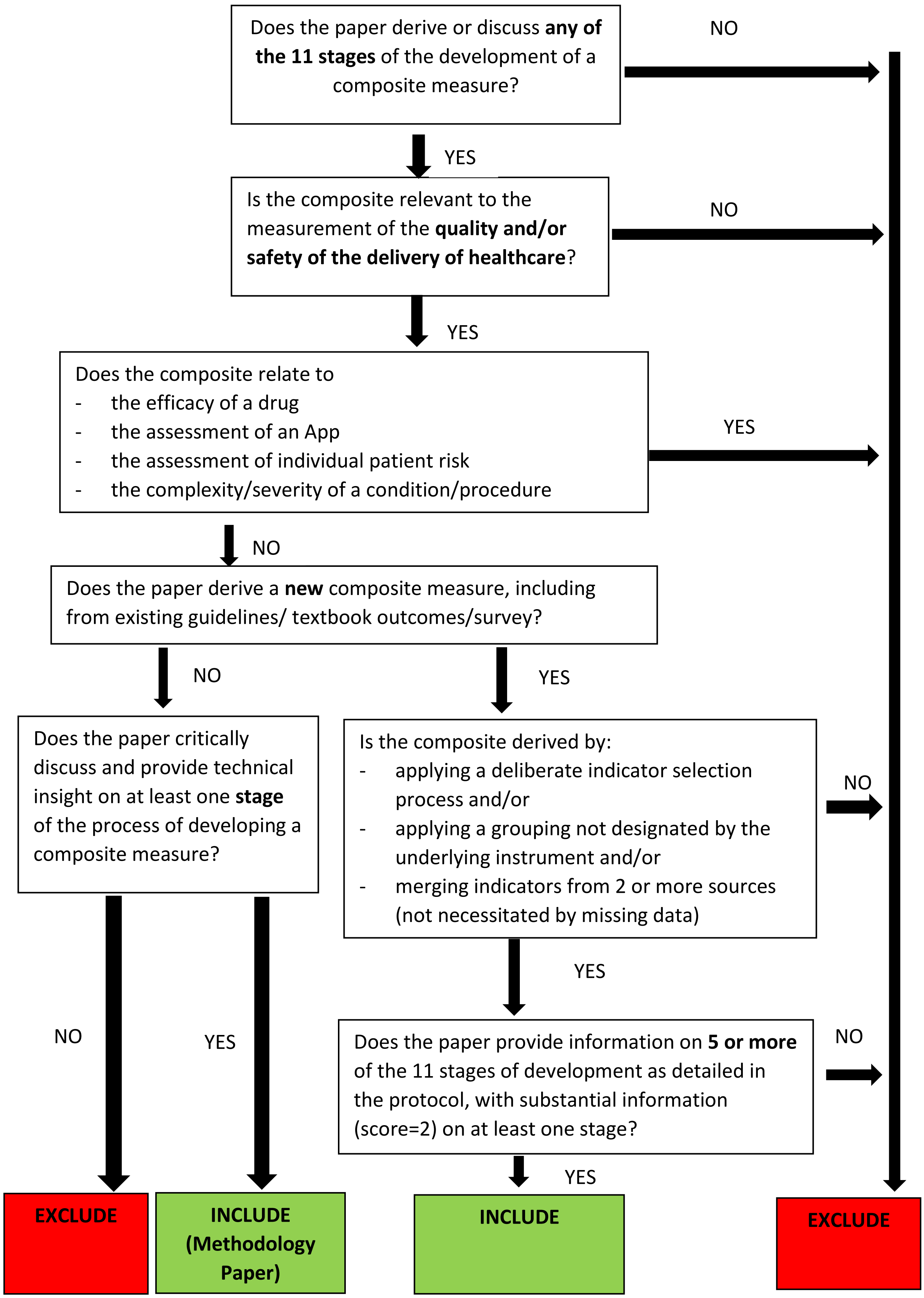

Supplement: Supplementary file 1 [file Supplementaryfile1.pdf]
