# Supplementary material for: Methods to derive composite indicators used for quality and safety measurement and monitoring in healthcare: a scoping review
Source: Front Health Serv. 2026 Aug 4;6:1852718. doi: 10.3389/frhs.2026.1852718 (PMC13481381; doi:10.3389/frhs.2026.1852718)
Supplement: Supplementary file 2 [file Supplementaryfile2.docx]

|  | **Supplementary File 2**  **Publications Developing Composite Measures**  **P1 – P8 Table 1 – List of Publications by Composite Classification**  **P9 Figure S1 – Publications development Composite Measures by Year (Jan 2000-Feb 2025)**  **P10 Figure S2 – Geographic Location of Studies (based on study data)**  **TABLE S1 Publications by Composite Classification** | | | |
| --- | --- | --- | --- | --- |
| **Ref** | **Lead Author & Year** | **Country** | **Composite Classification** | **Setting** |
| (1) | Agniel 2020 | United States | Process | Hospital(s) |
| (2) | Albright 2016 | United States | Process | Hospital(s) |
| (3) | Aliprandi-Costa 2017 | Australia | Process | Hospital(s) |
| (4) | Kukreja 2017 | United States | Process | Hospital(s) |
| (5) | Bachireddy 2014 | Other: Ukraine | Process | Community services |
| (6) | Bagonza 2014 | Other: Uganda | Process | Community services |
| (7) | Barnett 2017 | United States | Process | Hospital(s); General Practitioner |
| (8) | Beaulieu 2005 | United States | Process | General Practitioner |
| (9) | Bergholt 2021 | Other: Faroe Islands, Denmark | Process | Hospital(s) |
| (10) | Bethell 2004 | United States | Process | National or Regional level |
| (11) | Bintabara 2019 | Other: Tanzania | Process | Community services |
| (12) | Bogh 2015 | Other: Denmark | Process | Hospital(s) |
| (13) | Burmeister 2016 | Australia | Process | Hospital(s) |
| (14) | DeVore 2021 | UK | Process | Hospital(s) |
| (15) | De Wet 2012 | UK | Process | General Practitioner |
| (16) | Falstie-Jensen 2017 | Other: Denmark | Process | Hospital(s) |
| (17) | Garcia-Casanovas 2024 | Other: Spain | Process | Hospital(s) |
| (18) | Ghosh 2020 | India`` | Process | Hospital(s) |
| (19) | Granger 2022 | United States | Process | Hospital(s) |
| (20) | Guthrie 2011 | Other: Scotland | Process | General Practitioner |
| (21) | Halterman 2006 | United States | Process | General Practitioner |
| (22) | Hasegawa 2013 | Other: Japan | Process | Hospital(s) |
| (23) | Hastings 2007 | United States | Process | Hospital(s) |
| (24) | Hays 2014 | United States | Process | National or Regional level |
| (25) | Hepner 2019 | United States | Process | Community services |
| (26) | Ido 2018 | United States | Process | Hospital(s) |
| (27) | Iqbal 2017 | Other: Pakistan | Process | National or Regional level; Hospital(s); Community services |
| (28) | Jacobson 2008 | United States | Process | Community services |
| (29) | Jargalsaikhan 2025 | Other: Hungary | Process | General Practitioner |
| (30) | Khawagi 2022 | UK | Process | General Practitioner |
| (31) | Kilbourne 2010 | United States | Process | Other: Veteran mental health outpatients clinics |
| (32) | Kinnier 2015 | United States | Process | Hospital(s) |
| (33) | Kolfschoten 2012 | Other: Netherlands | Process | Hospital(s) |
| (34) | Kranz 2018 | United States | Process | Community services |
| (35) | Kumar 2013 | Other: India | Process | National or Regional level |
| (36) | Landon 2006 | United States | Process | Hospital(s) |
| (37) | Lee 2022 | Other: India | Process | National or Regional level |
| (38) | Li 2016 | Other: China | Process | Hospital(s) |
| (39) | Li 2021 | Other: China | Process | Hospital(s) |
| (40) | Liu 2024 | Other: China | Process | Hospital(s) |
| (41) | Lindenauer 2007 | United States | Process | Hospital(s); Home |
| (42, 43) | Mangione-Smith 2019 (also 2017 paper) | United States | Process | Hospital(s) |
| (44) | Mariz Batista 2022 | Other: Brazil | Process | Community services |
| (45) | Martín-Martínez 2019 | Other: Spain | Process | Hospital(s) |
| (46) | Martirosyan 2012 | Other: The Netherlands | Process | General Practitioner |
| (47) | Martsolf 2017 | United States | Process | General Practitioner |
| (48) | McHugh 2013 | United States | Process | Hospital(s) |
| (49) | Min 2005 | United States | Process | Community services |
| (50) | Murtas 2020 | Other: Italy | Process | Hospital(s) |
| (51) | Nass 2023 | Non-specific | Process | Hospital(s) |
| (52) | Navathe 2019 | United States | Process | General Practitioner |
| (53) | Nkoy 2015 | United States | Process | Hospital(s); Community services |
| (54) | Nutting 2007 | United States | Process | General Practitioner |
| (55) | Paustian 2014 | United States | Process | General Practitioner |
| (56) | Pinheiro 2022 | Other: Brazil | Process | Hospital(s); Community services |
| (57) | Reeves 2010 | United States | Process | Hospital(s) |
| (58) | Rodgers 2022 | UK | Process | General Practitioner |
| (59) | Rosner 2025 | United States | Process | Hospital(s) |
| (60) | Samuel 2015 | United States | Process | Hospital(s) |
| (61) | Schiele 2013 | Other: France | Process | National or Regional level; Hospital(s) |
| (62) | Schmajuk 2020 | United States | Process | Community services |
| (63) | Scholle 2008 | United States | Process | Other: Health plans |
| (64) | Schumacher 2020 | United States | Process | Hospital(s) |
| (65) | Segal 2015 | United States | Process | Hospital(s) |
| (66) | Simms 2013 | UK | Process | Hospital(s) |
| (67) | Smirnova 2022 | United States | Process | Hospital(s) |
| (68) | Spivack 2022 | United States | Process | General Practitioner |
| (69) | Starks 2018 | United States | Process | Hospital(s) |
| (70) | Stulberg 2010 | United States | Process | Hospital(s) |
| (71) | Thombley 2024 | United States | Process | Hospital(s) |
| (72) | Triyana 2017 | Other: Indonesia | Process | Community services |
| (73) | Tsai 2009 | United States | Process | Hospital(s) |
| (74) | Ukawa 2014 | Other: Japan | Process | Hospital(s) |
| (75) | Valladares 2022 | United States | Process | Hospital(s) |
| (76) | Van Roosmalen 2007 | Other: The Netherlands | Process | General Practitioner |
| (77) | Vichare 2013 | United States | Process | Hospital(s) |
| (78) | Wang 2018 | Other: China | Process | Hospital(s) |
| (79) | Wang 2020 | Other: China | Process | Hospital(s) |
| (80) | Weng 2015 | Canada | Process | Hospital(s) |
| (81) | Willis 2010 | UK & Australia | Process | Hospital(s) |
| (82) | Wu 2019 | Other: China | Process | Hospital(s) |
| (83) | Zeitlin 2016 | Europe | Process | Hospital(s) |
| (84) | Zerillo 2015 | United States | Process | Other: Oncology Practices |
| (85) | Zhang 2018 | Other: China | Process | Hospital(s) |
| (86) | Zuckerman 2004 | United States | Process | National or Regional level; Other: Assessed from parent survey |
| (87) | Alasfoor 2020 | Other: Oman | Outcome | Community services  /General Practitioner |
| (88) | Ayton 2020 | Australia | Outcome | Hospital(s) |
| (89) | Badhwar 2016 | United States | Outcome | Hospital(s) |
| (90) | Barbaree 2025 | Canada | Outcome | Hospital(s) |
| (91) | Beane 2021 | United States | Outcome | Hospital(s) |
| (92) | Boyle 2016 | United States | Outcome | Hospital(s) |
| (93) | Bozic 2014 | United States | Outcome | Hospital(s) |
| (94) | Broderick 2020 | United States | Outcome | Hospital(s) |
| (95) | Carey 2018 | United States | Outcome | Nursing home |
| (96) | Cartland 2022 | United States | Outcome | Hospital(s) |
| (97) | Caruso 2024 | United States | Outcome | Hospital(s) |
| (98) | Comart 2013 | United States | Outcome | Other: Long-term facility |
| (99) | Danilyants 2019 | United States | Outcome | Hospital(s) |
| (100) | Desai 2021 | United States | Outcome | Hospital(s) |
| (101) | Dimick 2013 | United States | Outcome | Hospital(s) |
| (102) | Dooley 2024 | United States | Outcome | Hospital(s) |
| (103) | Dunham 2018 | United States | Outcome | Hospital(s) |
| (104) | Eklom 2021 | Australia | Outcome | Hospital(s) |
| (105) | Glance 2019 | United States | Outcome | Hospital(s) |
| (106) | Gorgemans 2018 | Other: Spain | Outcome | Hospital(s) |
| (107) | Hardt 2019 | Other: Germany | Outcome | Hospital(s) |
| (108) | Hartman 2024 | United States | Outcome | Hospital(s) |
| (109) | Hermer 2018 | United States | Outcome | Nursing home |
| (110) | Hofstede 2019 | Other: Netherlands | Outcome | Hospital(s) |
| (111) | Hollenbeck 2020 | United States | Outcome | National or Regional level; Hospital(s) |
| (112) | Hyer 2022 | United States | Outcome | Hospital(s) |
| (113) | Kaempf 2015 | United States | Outcome | Hospital(s) |
| (114) | Karthaus 2017 | Other: Netherlands | Outcome | Hospital(s) |
| (115) | Keller 2014 | United States | Outcome | Hospital(s) |
| (116) | Kozower 2016 | United States | Outcome | Hospital(s) |
| (117) | Kuhrij 2020 | Other: Netherlands | Outcome | Hospital(s) |
| (118) | Lain 2012 | Australia | Outcome | Hospital(s)/National. |
| (119) | Lawson 2021 | Canada | Outcome | Hospital(s) |
| (120) | Li 2007 | United States | Outcome | Hospital(s) |
| (121) | Li 2021 | United States | Outcome | Hospital(s) |
| (122) | Lingsma 2018 | International | Outcome | Hospital(s) |
| (123) | Mann 2006 | International | Outcome | Hospital(s) |
| (124) | Mansfield 2015 | United States | Outcome | Hospital(s) |
| (125) | Merath 2020 | United States | Outcome | Hospital(s) |
| (126) | Milliren 2022 | United States | Outcome | Hospital(s) |
| (127) | Nair 2016 | UK | Outcome | Hospital(s) |
| (128) | Neary 2023 | Other: Ireland | Outcome | Hospital(s) |
| (129) | Numan 2016 | Other: The Netherlands | Outcome | Hospital |
| (130) | Pasquali 2019 | United States | Outcome | Hospital(s) |
| (131) | Patterson 2012 | United States | Outcome | Other: Other (Emergency Medical Services) |
| (132) | Pitocco 2022 | United States | Outcome | Nursing home |
| (133) | Poelemeijer 2019 | Other: Netherlands | Outcome | Hospital(s) |
| (134) | Ramani 2022 | United States | Outcome | Hospital(s) |
| (135) | Rankin 2017 | United States | Outcome | Hospital(s) |
| (136) | Reponen 2015 | Other: Finland | Outcome | Hospital(s) |
| (137) | Rosen 2009 | United States | Outcome | Hospital(s) |
| (138) | Rosen 2014 | United States | Outcome | Hospital(s) |
| (139) | Salet 2018 | Other: Netherlands | Outcome | Hospital(s) |
| (140) | Sammer 2020 | United States | Outcome | Hospital(s) |
| (141) | Seder 2017 | International | Outcome | National or Regional level; Hospital(s) |
| (142) | Shahian 2012 | United States | Outcome | Hospital(s) |
| (143) | Shahian 2014 | United States | Outcome | Hospital(s) |
| (144) | Shahian 2015 | United States | Outcome | Hospital(s) |
| (145) | Shahian 2022 | United States | Outcome | Hospital(s) |
| (146) | Shwartz 2014 | United States | Outcome | Nursing home |
| (147) | Smith 2019 | United States | Outcome | Hospital(s) |
| (148) | Smith 2025 | United States | Outcome | Hospital(s) |
| (149) | Straneo 2024 | Other: sub-Saharan Africa | Outcome | National or Regional level |
| (150) | Sullivan 2013 | United States | Outcome | Nursing home |
| (151) | Ten Berge 2021 | Other: Netherlands | Outcome | Hospital(s) |
| (152) | The STS 2017 | United States | Outcome | Hospital(s) |
| (153) | Van der Heide 2022 | Other: Netherlands | Outcome | Hospital(s) |
| (154) | Warps 2021 | Other: Netherlands | Outcome | Hospital(s) |
| (155) | Watt 2015 | UK | Outcome | Community services; General Practitioner; Home |
| (156) | Weaver 2012 | United States | Outcome | Community services; Other: Emergency Medical Services |
| (157) | Wiseman 2020 | United States | Outcome | Hospital(s) |
| (158) | Wiseman 2021 | United States | Outcome | Hospital(s) |
| (159) | Zaslavsky 2002 | United States | Outcome | Hospital(s); Community services; General Practitioner; Other: Healthcare Providers (Plan) |
| (160) | Zogg 2023 | United States | Outcome | Hospital(s) |
| (161) | Aaronson 2013 | United States | Mix | Hospital(s) |
| (162) | Agniel 2021 | United States | Mix | National or Regional level |
| (163) | Allorant 2020 | Other: Haiti | Mix | Hospital(s); Community services; Other: Health Facility |
| (164) | Amoah 2015 | United States | Mix | General Practitioner |
| (165) | Anthony 2004 | United States | Mix | Hospital(s) |
| (166) | Aoki 2023 | Other: Angola | Mix | Hospital(s); Community services |
| (167) | Aragon 2022 | United States | Mix | Hospital(s) |
| (168) | Ashby 2012 | United States | Mix | Hospital(s) |
| (169) | Austin 2014 | United States | Mix | Hospital(s) |
| (170) | Bilimoria 2009 | United States | Mix | Hospital(s) |
| (171) | Bilimoria 2009 | United States | Mix | Hospital(s) |
| (172) | Brewer 2013 | United States | Mix | Hospital(s) |
| (173) | Brown 2019 | United States | Mix | Hospital(s) |
| (174) | Brunelli 2009 | Other: Europe | Mix | Hospital(s) |
| (175) | Brunelli 2010 | Other: Europe | Mix | Hospital(s) |
| (176) | Bruzzi 2022 | Other: Italy | Mix | National or Regional level |
| (177) | Cabrera-Barona 2017 | Other: Quito, Ecuador. | Mix | National or Regional level |
| (178) | Calciolari 2016 | Other: Italy | Mix | National or Regional level |
| (179) | Calderon-Margalit 2018 | Other: Israel | Mix | Community services |
| (180) | Carey 2021 | United States | Mix | Community services |
| (181) | Chen 2012 | Other: Taiwan | Mix | Hospital(s) |
| (182) | Chen 2013 | United States | Mix | Hospital(s) |
| (183) | Choi 2022 | United States | Mix | National or Regional level |
| (184) | Damberg 2010 | United States | Mix | Hospital(s) |
| (185) | Katz 2020 | United States | Mix | Other: Veteran |
| (186) | Dehanne 2024 | Other: Belgium | Mix | National or Regional level; Hospital(s) |
| (187) | de Miranda Costa 2020 | Other: Brazil | Mix | Hospital(s) |
| (188) | de Oliveira Valentino 2020 | Other: Brazil | Mix | Hospital(s) |
| (189) | Dhillon 2020 | Other: India | Mix | Hospital(s) |
| (190) | Dimick 2009 | United States | Mix | Hospital(s) |
| (191) | Dimick 2012 | United States | Mix | Hospital(s) |
| (192) | Dimick 2014 | United States | Mix | Hospital(s) |
| (193) | Dong 2019 | Other: China | Mix | Hospital(s) |
| (194) | Duggal 2022 | Other: India | Mix | Hospital(s) |
| (195) | Dusheiko 2015 | UK | Mix | General Practitioner |
| (196) | Ekouevi 2012 | Other: South Africa, Zambia, Cameroon, Cote d'Ivoire | Mix | Hospital(s) |
| (197) | Ferreira 2020 | Other: Portugal | Mix | Hospital(s) |
| (198) | Fieldston 2014 | United States | Mix | Hospital(s) |
| (199) | Fong 2008 | United States | Mix | Hospital(s) |
| (200) | Fruhauf 2018 | Other: East Africa | Mix | Community services |
| (201) | Gage 2017 | Other: Haiti | Mix | Community services |
| (202) | Gage 2018 | Other: Haiti | Mix | Community services |
| (203) | Gajewski 2013 | United States | Mix | Hospital(s) |
| (204) | Garrido-Cumbrera 2008 | Other: Spain | Mix | National or Regional level |
| (205) | Getachew 2020 | Other: East Africa | Mix | Hospital(s) |
| (206) | Goh 2023 | Australia | Mix | Hospital(s) |
| (207) | Hamad 2024 | United States | Mix | Hospital(s) |
| (208) | Hess 2011 | United States | Mix | Other: Setting not specified: Clinical practice |
| (209) | Hicks 2010 | United States | Mix | Community services |
| (210) | Holbrook 2009 | United States | Mix | General Practitioner |
| (211) | Holmboe 2010 | United States | Mix | General Practitioner |
| (212) | Holmboe 2010 | United States | Mix | General Practitioner |
| (213) | Horberg 2020 | United States | Mix | Other: Other (Integrated Health System) |
| (214) | Horwood 2019 | Other: South Africa | Mix | Hospital(s) |
| (215) | Hota 2020 | United States | Mix | Hospital(s) |
| (216) | Iyengar 2019 | United States | Mix | Hospital(s) |
| (217) | Jain 2018 | United States | Mix | National or Regional level |
| (218) | Kadoić 2021 | Other: Croatia | Mix | Hospital(s) |
| (219) | Kane 2007 | United States | Mix | Nursing home |
| (220) | Kaplan 2009 | United States | Mix | Other: Doctor level: primary care, endocrinology, multilpe-speciality practices. |
| (221) | Katz 2017 | Canada | Mix | Community services |
| (222) | Kumar 2021 | Other: India | Mix | Hospital(s) |
| (223) | Lalloué 2017 | Other: France | Mix | Hospital(s) |
| (224) | Landon 2007 | United States | Mix | Community services |
| (225) | Landrum 2000 | United States | Mix | Hospital(s) |
| (226) | Lawson 2017 | United States | Mix | Hospital(s) |
| (227) | Leegwater 2015 | International | Mix | National or Regional level |
| (228) | Lemke 2017 | United States | Mix | Hospital(s) |
| (229) | Levine 2011 | United States | Mix | Community services |
| (230) | Lied 2002 | United States | Mix | National or Regional level; Other: Health Care Plans |
| (231) | Lipner 2013 | United States | Mix | General Practitioner |
| (232) | Liu 2013 | United States | Mix | Hospital(s) |
| (233) | Portela 2016 | Other: Portugal | Mix | Hospital(s) |
| (234) | Matos 2021 | Other: Portugal | Mix | Hospital(s) |
| (235) | Mays 2004 | United States | Mix | National or Regional level |
| (236) | Megwalu 2020 | United States | Mix | Hospital(s) |
| (237) | Modi 2019 | Other: India | Mix | Community services |
| (238) | Monsees 2020 | United States | Mix | Hospital(s) |
| (239) | Mulder 2022 | Canada | Mix | Community services |
| (240) | Nelson 2014 | United States | Mix | Hospital(s); Community services |
| (241) | Normand 2022 | United States | Mix | Hospital(s); Community services |
| (242) | Otieno 2008 | Other: Japan | Mix | Hospital(s) |
| (243) | Oluwatola 2025 | Other: Nigeria | Mix | Hospital(s) |
| (244) | Pakyz 2014 | United States | Mix | Hospital(s) |
| (245) | Pedroja 2014 | United States | Mix | Hospital(s) |
| (246) | Peters 2007 | Other: Afghanistan | Mix | National or Regional level |
| (247) | Popescu 2011 | United States | Mix | Hospital(s) |
| (248) | Prinja 2017 | Other: India | Mix | National or Regional level |
| (249) | Profit 2014 | United States | Mix | Hospital(s) |
| (250) | Rhee 2018 | Other: African Region | Mix | National or Regional level; Hospital(s); Home |
| (251) | Ross 2011 | United States | Mix | Hospital(s) |
| (252) | Ryan 2017 | United States | Mix | Hospital(s) |
| (253) | Scaratti 2012 | Other: Brazil | Mix | Community services |
| (254) | Sequist 2008 | United States | Mix | General Practitioner |
| (255) | Sequist 2012 | United States | Mix | Hospital(s); General Practitioner |
| (256) | Shahian 2007 | United States | Mix | Hospital(s) |
| (257) | Sharma 2019 | Other: India | Mix | National or Regional level; Hospital(s); Community services |
| (258) | Shwartz 2011 | United States | Mix | Hospital(s) |
| (259) | Silva 2014 | Other: Brazil | Mix | Hospital(s) |
| (260) | Staiger 2009 | United States | Mix | Hospital(s) |
| (261) | Tardivo 2018 | Other: Italy | Mix | Hospital(s) |
| (262) | Testa 2024 | Other: Italy | Mix | National or Regional level |
| (263) | Timbie 2009 | United States | Mix | Hospital(s) |
| (264) | Tsai 2015 | United States | Mix | Hospital(s) |
| (265) | Urick 2019 | United States | Mix | Community services |
| (266) | Van Doorn-Klomberg 2014 | Other: The Netherlands | Mix | General Practitioner |
| (267) | Vieira-Meyer 2020 | Other: Brazil | Mix | National or Regional level |
| (268) | Weeks 2016 | United States | Mix | Hospital(s) |
| (269) | Weifeng 2010 | United States | Mix | General Practitioner; Nursing home |
| (270) | Wilberforce 2011 | UK | Mix | Community services |
| (271) | Wilhelm 2019 | Other: Malawi | Mix | Hospital(s) |
| (272) | Yan 2018 | Other: Zambia | Mix | Hospital(s); Community services |
| (273) | Anhang Price 2018 | United States | Experience | Other: Hospice |
| (274) | Anhang Price 2022 | United States | Experience | Home |
| (275) | Antoniotti 2009 | Other: France | Experience | Hospital(s) |
| (276) | Asan 2021 | United States | Experience | Other: Clinician |
| (277) | Berger 2021 | Other: Nigeria, Ghana and Guinea | Experience | Hospital(s) |
| (278) | Brown 2021 | United States | Experience | Hospital(s)/Clinics/Regional |
| (279) | Callegari 2022 | United States | Experience | Community services |
| (280) | Clarke-Deelder 2022 | Other: Tanzania | Experience | Community services |
| (281) | Davis 2016 | United States | Experience | Hospital(s) |
| (282) | Desai 2020 | United States | Experience | Hospital(s) |
| (283) | Dixon 2016 | United States | Experience | General Practitioner |
| (284) | Evensen 2019 | United States | Experience | Hospital(s) |
| (285) | Gomez-Cano 2022 | UK | Experience | Other: Cancer care - across various settings |
| (286) | Hargraves 2003 | United States | Experience | Community services; General Practitioner |
| (287) | Hotchkiss 2022 | United States | Experience | Other: Other (Hospice care) |
| (288) | Jensen 2017 | Denmark and the Netherlands | Experience | Hospital(s) |
| (289) | Johnson 2021 | United States | Experience | Hospital(s) |
| (290) | Keller 2009 | United States | Experience | Other: Dental Surgery |
| (291) | Kranz 2020 | United States | Experience | Community services |
| (292) | Langford 2019 | United States | Experience | National or Regional level |
| (293) | Larson 2014 | Other: Tanzania | Experience | Community services |
| (294) | Lim 2021 | Other: Malaysia | Experience | General Practitioner |
| (295) | Makary 2006 | United States | Experience | Hospital(s) |
| (296) | Milliken-Glabe 2017 | United States | Experience | Hospital(s) |
| (297) | Minvielle 2005 | Other: France | Experience | Hospital(s) |
| (298) | Olaisen 2019 | United States | Experience | General Practitioner |
| (299) | O'Malley 2005 | Canada | Experience | Hospital(s) |
| (300) | Ragavan 2020 | United States | Experience | Hospital(s) |
| (301) | Reszel 2021 | Canada | Experience | Community services; Hospital(s) |
| (302) | Ruiz 2008 | Other: Spain | Experience | Hospital(s); Community services; Nursing home |
| (303) | Sawicki 2015 | United States | Experience | Hospital(s) |
| (304) | Sequist 2009 | United States | Experience | General Practitioner |
| (305) | Solberg 2014 | United States | Experience | General Practitioner |
| (306) | Sorra 2021 | United States | Experience | Hospital(s); Home |
| (307) | Sorra 2022 | United States | Experience | Hospital(s); General Practitioner |
| (308) | Teruya 2022 | United States | Experience | Community services |
| (309) | Toomey 2015 | United States | Experience | Hospital(s) |
| (310) | Tuladhar 2024 | Other: Nepal | Experience | Hospital(s) |
| (311) | Wahl 2020 | Other: Sweden | Experience | Hospital |
| (312) | Weidmer 2012 | United States | Experience | Community services |
| (313) | Wiegers 2009 | Other: Netherlands | Experience | National or Regional level; Hospital(s); Community services; General Practitioner; Home |
| (314) | Wojcik 2019 | United States | Experience | Hospital(s) |
| (315) | Ye 2022 | United States | Experience | Hospital(s) |
| (316) | Zebrak 2022 | United States | Experience | Hospital(s) |
| (317) | Amadeo 2011 | Other: France | Structural | Hospital(s) |
| (318) | Aggarwal 2023 | Other: India | Structural | Hospital(s) |
| (319) | Das 2022 | Other: India | Structural | National or Regional level |
| (320) | Gupta 2023 | United States | Structural | National or Regional level |
| (321) | Henry 2017 | Other: Zambia | Structural | Other: Maternity waiting homes |
| (322) | Iyer 2015 | Other: Rwanda | Structural | Hospital(s) |
| (323) | Pelly 2021 | Other: India | Structural | Community services |
| (324) | Powell-Jackson 2013 | Other:India | Structural | Community services |
| (325) | Reddy 2020 | Other: Ireland | Structural | Nursing home |
| (326) | Shin 2012 | Other: Korea | Structural | Hospital(s) |
|  |  |  |  |  |
|  |  |  |  |  |
|  |  |  |  |  |

**Figure S1: Distribution of Information Score by 10 stages.**

Information Score by Stage

Papers

Mean score

|  | 0 | 1 | 2 | mean |
| --- | --- | --- | --- | --- |
| Framework | 95 | 179 | 51 | 0.864615 |
| Indicator Selection | 1 | 147 | 177 | 1.54154 |
| Data analysis | 142 | 49 | 275 | 1.84308 |
| Missing Data | 142 | 128 | 55 | 0.732308 |
| Normalisation | 7 | 210 | 108 | 1.31077 |
| Weighting & Aggregation | 0 | 201 | 124 | 1.38154 |
| Uncertainty | 147 | 115 | 63 | 0.741538 |
| Linkage | 56 | 92 | 177 | 1.37231 |
| Deconstruction | 24 | 108 | 193 | 1.52 |
| Presentation | 12 | 153 | 160 | 1.45538 |

**Figure S2 – Publications development Composite Measures by Year (Jan 2000-Feb 2025)**

*(note: 2025 is 2 months only)*

Publications by Year

|  | Total |
| --- | --- |
| 2000 | 1 |
| 2002 | 2 |
| 2003 | 1 |
| 2004 | 4 |
| 2005 | 4 |
| 2006 | 4 |
| 2007 | 9 |
| 2008 | 7 |
| 2009 | 14 |
| 2010 | 10 |
| 2011 | 8 |
| 2012 | 15 |
| 2013 | 16 |
| 2014 | 22 |
| 2015 | 18 |
| 2016 | 14 |
| 2017 | 23 |
| 2018 | 20 |
| 2019 | 25 |
| 2020 | 30 |
| 2021 | 24 |
| 2022 | 31 |
| 2023 | 7 |
| 2024 | 11 |
| 2025 | 5 |

**Figure S3 – Geographic Location of Studies (based on study data)**

Geographic Location of Study

|  |  |
| --- | --- |
| United States | 185 |
| Europe | 50 |
| Asia | 20 |
| Africa | 17 |
| India | 13 |
| South America | 10 |
| Australia | 7 |
| Canada | 7 |
| International | 5 |
| UK | 11 |

**Note:** *Number of papers by country (n>5). Grouped by continent for those with a smaller number of papers. Designated as International where the country is not specified or data relates to a number of countries across continents.*

**REFERENCES:**

1.	Agniel D, Haviland A, Shekelle P, Scherling A, Damberg CL. Distinguishing High-Performing Health Systems Using a Composite of Publicly Reported Measures of Ambulatory Care. Ann Intern Med. 2020;173(10):791-8.

2.	Albright BB, Lewis VA, Ross JS, Colla CH. Preventive Care Quality of Medicare Accountable Care Organizations: Associations of Organizational Characteristics With Performance. Med Care. 2016;54(3):326-35.

3.	Aliprandi-Costa B, Sockler J, Kritharides L, Morgan L, Snell LC, Gullick J, et al. The contribution of the composite of clinical process indicators as a measure of hospital performance in the management of acute coronary syndromes-insights from the CONCORDANCE registry. Eur Heart J Qual Care Clin Outcomes. 2017;3(1):37-46.

4.	Baack Kukreja JE, Kiernan M, Schempp B, Siebert A, Hontar A, Nelson B, et al. Quality Improvement in Cystectomy Care with Enhanced Recovery (QUICCER) study. BJU Int. 2017;119(1):38-49.

5.	Bachireddy C, Soule MC, Izenberg JM, Dvoryak S, Dumchev K, Altice FL. Integration of health services improves multiple healthcare outcomes among HIV-infected people who inject drugs in Ukraine. Drug Alcohol Depend. 2014;134:106-14.

6.	Bagonza J, Kibira SP, Rutebemberwa E. Performance of community health workers managing malaria, pneumonia and diarrhoea under the community case management programme in central Uganda: a cross sectional study. Malar J. 2014;13:367.

7.	Barnett ML, Linder JA, Clark CR, Sommers BD. Low-Value Medical Services in the Safety-Net Population. JAMA Intern Med. 2017;177(6):829-37.

8.	Beaulieu ND, Horrigan DR. Putting smart money to work for quality improvement. Health Serv Res. 2005;40(5 Pt 1):1318-34.

9.	Bergholt MD, Falstie-Jensen AM, Hibbert P, Eysturoy BJ, Guttesen G, Róin T, et al. The association between first-time accreditation and the delivery of recommended care: a before and after study in the Faroe Islands. BMC Health Serv Res. 2021;21(1):917.

10.	Bethell C, Reuland CH, Halfon N, Schor EL. Measuring the quality of preventive and developmental services for young children: national estimates and patterns of clinicians' performance. Pediatrics. 2004;113(6 Suppl):1973-83.

11.	Bintabara D, Nakamura K, Ntwenya J, Seino K, Mpondo BCT. Adherence to standards of first-visit antenatal care among providers: A stratified analysis of Tanzanian facility-based survey for improving quality of antenatal care. PLoS One. 2019;14(5):e0216520.

12.	Bogh SB, Falstie-Jensen AM, Bartels P, Hollnagel E, Johnsen SP. Accreditation and improvement in process quality of care: a nationwide study. Int J Qual Health Care. 2015;27(5):336-43.

13.	Burmeister EA, O'Connell DL, Jordan SJ, Goldstein D, Merrett N, Wyld DK, et al. Factors associated with quality of care for patients with pancreatic cancer in Australia. Med J Aust. 2016;205(10):459-65.

14.	DeVore AD, Granger BB, Fonarow GC, Al-Khalidi HR, Albert NM, Lewis EF, et al. Effect of a Hospital and Postdischarge Quality Improvement Intervention on Clinical Outcomes and Quality of Care for Patients With Heart Failure With Reduced Ejection Fraction: The CONNECT-HF Randomized Clinical Trial. Jama. 2021;326(4):314-23.

15.	de Wet C, McKay J, Bowie P. Combining QOF data with the care bundle approach may provide a more meaningful measure of quality in general practice. BMC Health Services Research. 2012;12:351.

16.	Falstie-Jensen AM, Bogh SB, Hollnagel E, Johnsen SP. Compliance with accreditation and recommended hospital care-a Danish nationwide population-based study. Int J Qual Health Care. 2017;29(5):625-33.

17.	Garcia-Casanovas A, Bisbe E, Garcia-Altes A, Vizoso A, Duran-Jorda X, Sanchez-Pedrosa G, et al. Hospital variation in quality indicators for patient blood management in total knee and hip arthroplasty: a retrospective cohort study. Br J Anaesth. 2024;133(3):637-46.

18.	Ghosh R, Spindler H, Dyer J, Christmas A, Cohen SR, Das A, et al. Simulation and team training embedded nurse mentoring programme and improvement in intrapartum and newborn care in a low-resource setting in Bihar, India. J Glob Health. 2020;10(2):021010.

19.	Granger B, Devore A, Kaltenbach L, Fonarow G, Al-Khalidi H, Albert N, et al. Performance On Guideline Directed Medical Therapy Remains Low In A Cluster-randomized Trial: Results From CONNECT-HF. Journal of Cardiac Failure. 2022;28(5):S43.

20.	Guthrie B, McCowan C, Davey P, Simpson CR, Dreischulte T, Barnett K. High risk prescribing in primary care patients particularly vulnerable to adverse drug events: cross sectional population database analysis in Scottish general practice. Bmj. 2011;342:d3514.

21.	Halterman JS, Kitzman H, McMullen A, Lynch K, Fagnano M, Conn KM, et al. Quantifying preventive asthma care delivered at office visits: The preventive asthma care-composite index (PAC-CI). Journal of Asthma. 2006;43(7):559-64.

22.	Hasegawa K, Chiba T, Hagiwara Y, Watase H, Tsugawa Y, Brown DF, et al. Quality of care for acute asthma in emergency departments in Japan: a multicenter observational study. J Allergy Clin Immunol Pract. 2013;1(5):509-15.e1-3.

23.	Hastings SN, Sloane RJ, Goldberg KC, Oddone EZ, Schmader KE. The quality of pharmacotherapy in older veterans discharged from the emergency department or urgent care clinic. J Am Geriatr Soc. 2007;55(9):1339-48.

24.	Hays RD, Martino S, Brown JA, Cui M, Cleary P, Gaillot S, et al. Evaluation of a Care Coordination Measure for the Consumer Assessment of Healthcare Providers and Systems (CAHPS) Medicare survey. Med Care Res Rev. 2014;71(2):192-202.

25.	Hepner KA, Paddock SM, Watkins KE, Hoggatt KJ, Rubenstein LV, Bogart A, et al. Rates and impact of adherence to recommended care for unhealthy alcohol use. Journal of general internal medicine. 2019;34:256-63.

26.	Ido MS, Frankel MR, Okosun IS, Rothenberg RB. Quality of Care and Its Impact on One-Year Mortality: The Georgia Coverdell Acute Stroke Registry. Am J Med Qual. 2018;33(1):86-92.

27.	Iqbal S, Maqsood S, Zakar R, Zakar MZ, Fischer F. Continuum of care in maternal, newborn and child health in Pakistan: analysis of trends and determinants from 2006 to 2012. BMC Health Serv Res. 2017;17(1):189.

28.	Jacobson JO, Neuss MN, McNiff KK, Kadlubek P, Thacker LR, 2nd, Song F, et al. Improvement in oncology practice performance through voluntary participation in the Quality Oncology Practice Initiative. J Clin Oncol. 2008;26(11):1893-8.

29.	Jargalsaikhan U, Kasabji F, Vincze F, Pálinkás A, Kőrösi L, Sándor J. A Composite Indicator for Primary Diabetes Care: A Cross-Sectional Study in Hungary. Healthcare (Basel). 2025;13(5).

30.	Khawagi WY, Steinke D, Carr MJ, Wright AK, Ashcroft DM, Avery A, et al. Evaluating the safety of mental health-related prescribing in UK primary care: a cross-sectional study using the Clinical Practice Research Datalink (CPRD). BMJ Qual Saf. 2022;31(5):364-78.

31.	Kilbourne AM, Farmer Teh C, Welsh D, Pincus HA, Lasky E, Perron B, et al. Implementing composite quality metrics for bipolar disorder: towards a more comprehensive approach to quality measurement. Gen Hosp Psychiatry. 2010;32(6):636-43.

32.	Kinnier CV, Ju MH, Kmiecik T, Barnard C, Halverson T, Yang AD, et al. Development of a Novel Composite Process Measure for Venous Thromboembolism Prophylaxis. Med Care. 2016;54(2):210-7.

33.	Kolfschoten NE, Gooiker GA, Bastiaannet E, van Leersum NJ, van de Velde CJ, Eddes EH, et al. Combining process indicators to evaluate quality of care for surgical patients with colorectal cancer: are scores consistent with short-term outcome? BMJ Qual Saf. 2012;21(6):481-9.

34.	Kranz AM, Dalton S, Damberg C, Timbie JW. Using Health IT to Coordinate Care and Improve Quality in Safety-Net Clinics. Jt Comm J Qual Patient Saf. 2018;44(12):731-40.

35.	Kumar C, Singh PK, Rai RK. Coverage gap in maternal and child health services in India: assessing trends and regional deprivation during 1992-2006. J Public Health (Oxf). 2013;35(4):598-606.

36.	Landon BE, Normand SL, Lessler A, O'Malley AJ, Schmaltz S, Loeb JM, et al. Quality of care for the treatment of acute medical conditions in US hospitals. Arch Intern Med. 2006;166(22):2511-7.

37.	Lee HY, Rana MJ, Kim R, Subramanian SV. Small Area Variation in the Quality of Maternal and Newborn Care in India. JAMA Netw Open. 2022;5(11):e2242666.

38.	Li Z, Wang C, Zhao X, Liu L, Wang C, Li H, et al. Substantial Progress Yet Significant Opportunity for Improvement in Stroke Care in China. Stroke. 2016;47(11):2843-9.

39.	Li J, Qu P, Wang C, Li X, Hou S, Liu M. Quality-of-care comparison of stroke: The reliability and robustness of ranking by process or outcome measures. Int J Stroke. 2021:17474930211053139.

40.	Liu M, Guo R, Li J, Wang C, Yu L, Liu M. Process indicators outshine outcome measures: assessing hospital quality of care in breast cancer treatment in China. Sci Rep. 2024;14(1):19137.

41.	Lindenauer PK, Remus D, Roman S, Rothberg MB, Benjamin EM, Ma A, et al. Public reporting and pay for performance in hospital quality improvement. N Engl J Med. 2007;356(5):486-96.

42.	Mangione-Smith R, Roth CP, Britto MT, Chen AY, McGalliard J, Boat TF, et al. Development and Testing of the P ediatric R espiratory I llness Me asurement S ystem (PRIMES) Quality Indicators. Hospital Pediatrics. 2017;7(3):125-33.

43.	Mangione-Smith R, Zhou C, Williams DJ, Johnson DP, Kenyon CC, Tyler A, et al. Pediatric Respiratory Illness Measurement System (PRIMES) Scores and Outcomes. Pediatrics. 2019;144(2).

44.	Mariz Batista A, da Silva Gama ZA, Souza D. Validation of the QualiPresc instrument for assessing the quality of drug prescription writing in primary health care. PLoS One. 2022;17(5):e0267707.

45.	Martín-Martínez MA, Andreu-Sanchez JL, Sanchez-Alonso F, Corominas H, Perez-Venegas JJ, Roman-Ivorra JA, et al. A composite indicator to assess the quality of care in the management of patients with rheumatoid arthritis in outpatient rheumatology clinics. Reumatol Clin (Engl Ed). 2019;15(3):156-64.

46.	Martirosyan L, Haaijer-Ruskamp FM, Braspenning J, Denig P. Development of a minimal set of prescribing quality indicators for diabetes management on a general practice level. Pharmacoepidemiol Drug Saf. 2012;21(10):1053-9.

47.	Martsolf GR, Carle AC, Scanlon DP. Creating Unidimensional Global Measures of Physician Practice Quality Based on Health Insurance Claims Data. Health Serv Res. 2017;52(3):1061-78.

48.	McHugh M, Neimeyer J, Powell E, Khare RK, Adams JG. An early look at performance on the emergency care measures included in Medicare's hospital inpatient Value-Based Purchasing Program. Ann Emerg Med. 2013;61(6):616-23.e2.

49.	Min LC, Reuben DB, MacLean CH, Shekelle PG, Solomon DH, Higashi T, et al. Predictors of overall quality of care provided to vulnerable older people. J Am Geriatr Soc. 2005;53(10):1705-11.

50.	Murtas R, Decarli A, Greco MT, Andreano A, Russo AG. Latent composite indicators for evaluating adherence to guidelines in patients with a colorectal cancer diagnosis. Medicine (Baltimore). 2020;99(8):e19277.

51.	Nass KJ, van Doorn SC, Fockens P, Rees CJ, Pellisé M, van der Vlugt M, et al. High quality colonoscopy: using textbook process as a composite quality measure. Endoscopy. 2023;55(9):812-9.

52.	Navathe AS, Emanuel EJ, Bond A, Linn K, Caldarella K, Troxel A, et al. Association Between the Implementation of a Population-Based Primary Care Payment System and Achievement on Quality Measures in Hawaii. Jama. 2019;322(1):57-68.

53.	Nkoy F, Fassl B, Stone B, Uchida DA, Johnson J, Reynolds C, et al. Improving Pediatric Asthma Care and Outcomes Across Multiple Hospitals. Pediatrics. 2015;136(6):e1602-10.

54.	Nutting PA, Dickinson WP, Dickinson LM, Nelson CC, King DK, Crabtree BF, et al. Use of chronic care model elements is associated with higher-quality care for diabetes. Ann Fam Med. 2007;5(1):14-20.

55.	Paustian ML, Alexander JA, El Reda DK, Wise CG, Green LA, Fetters MD. Partial and incremental PCMH practice transformation: implications for quality and costs. Health Serv Res. 2014;49(1):52-74.

56.	Pinheiro JMF, Costa K, Lyra CO, Dos Santos F, Vianna RPT, Silva KL, et al. Care actions for newborns and factors associated with longitudinality in the follow-up care in the neonatal period: Care actions for the newborn. Medicine (Baltimore). 2022;101(45):e31500.

57.	Reeves MJ, Gargano J, Maier KS, Broderick JP, Frankel M, LaBresh KA, et al. Patient-level and hospital-level determinants of the quality of acute stroke care: a multilevel modeling approach. Stroke. 2010;41(12):2924-31.

58.	Rodgers S, Taylor AC, Roberts SA, Allen T, Ashcroft DM, Barrett J, et al. Scaling-up a pharmacist-led information technology intervention (PINCER) to reduce hazardous prescribing in general practices: Multiple interrupted time series study. PLoS Med. 2022;19(11):e1004133.

59.	Rosner B, Thombley R, Rogers S, Adler Milstein J. 4 Steps to 4Ms: A Navigation Guide for a Hospital-Based Composite Measure of 4Ms Care and the Implications for Outcomes Assessment. Inquiry. 2025;62:469580251323135.

60.	Samuel CA, Zaslavsky AM, Landrum MB, Lorenz K, Keating NL. Developing and Evaluating Composite Measures of Cancer Care Quality. Med Care. 2015;53(1):54-64.

61.	Schiele F, Capuano F, Loirat P, Desplanques-Leperre A, Derumeaux G, Thebaut JF, et al. Hospital case volume and appropriate prescriptions at hospital discharge after acute myocardial infarction: a nationwide assessment. Circ Cardiovasc Qual Outcomes. 2013;6(1):50-7.

62.	Schmajuk G, Li J, Evans M, Anastasiou C, Izadi Z, Kay JL, et al. RISE registry reveals potential gaps in medication safety for new users of biologics and targeted synthetic DMARDs. Seminars in Arthritis & Rheumatism. 2020;50(6):1542-8.

63.	Scholle SH, Roski J, Adams JL, Dunn DL, Kerr EA, Dugan DP, et al. Benchmarking physician performance: reliability of individual and composite measures. Am J Manag Care. 2008;14(12):833-8.

64.	Schumacher DJ, Martini A, Holmboe E, Carraccio C, van der Vleuten C, Sobolewski B, et al. Initial Implementation of Resident-Sensitive Quality Measures in the Pediatric Emergency Department: A Wide Range of Performance. Acad Med. 2020;95(8):1248-55.

65.	Segal JB, Nassery N, Chang H-Y, Chang E, Chan K, Bridges JF. An index for measuring overuse of health care resources with Medicare claims. Medical care. 2015;53(3):230-6.

66.	Simms AD, Baxter PD, Cattle BA, Batin PD, Wilson JI, West RM, et al. An assessment of composite measures of hospital performance and associated mortality for patients with acute myocardial infarction. Analysis of individual hospital performance and outcome for the National Institute for Cardiovascular Outcomes Research (NICOR). Eur Heart J Acute Cardiovasc Care. 2013;2(1):9-18.

67.	Smirnova A, Chahine S, Milani C, Schuh A, Sebok-Syer SS, Swartz J, et al. Using Resident-Sensitive Quality Measures Derived From Electronic Health Record Data to Assess Residents' Performance in Pediatric Emergency Medicine. Acad Med. 2022.

68.	Spivack SB, DeWalt D, Oberlander J, Trogdon J, Shah N, Meara E, et al. The Association of Readmission Reduction Activities with Primary Care Practice Readmission Rates. J Gen Intern Med. 2022;37(12):3005-12.

69.	Starks MA, Dai D, Nichol G, Al-Khatib SM, Chan P, Bradley SM, et al. The Association of Duration of participation in get with the guidelines-resuscitation with quality of Care for in-Hospital Cardiac Arrest. Am Heart J. 2018;204:156-62.

70.	Stulberg JJ, Delaney CP, Neuhauser DV, Aron DC, Fu P, Koroukian SM. Adherence to surgical care improvement project measures and the association with postoperative infections. Jama. 2010;303(24):2479-85.

71.	Thombley RL, Rogers SE, Adler-Milstein J. Developing electronic health record-based measures of the 4Ms to support implementation and evidence generation for Age-Friendly Health Systems. J Am Geriatr Soc. 2024;72(3):882-91.

72.	Triyana M, Shankar AH. The effects of a household conditional cash transfer programme on coverage and quality of antenatal care: a secondary analysis of Indonesia's pilot programme. BMJ Open. 2017;7(10):e014348.

73.	Tsai C-L, Sullivan AF, Gordon JA, Kaushal R, Magid DJ, Blumenthal D, et al. Quality of care for acute asthma in 63 US emergency departments. Journal of allergy and clinical immunology. 2009;123(2):354-61.

74.	Ukawa N, Ikai H, Imanaka Y. Trends in hospital performance in acute myocardial infarction care: a retrospective longitudinal study in Japan. Int J Qual Health Care. 2014;26(5):516-23.

75.	Valladares AF, McCauley SM, Khan M, D'Andrea C, Kilgore K, Mitchell K. Development and Evaluation of a Global Malnutrition Composite Score. J Acad Nutr Diet. 2022;122(2):251-8.

76.	van Roosmalen MS, Braspenning JC, De Smet PA, Grol RP. Antibiotic prescribing in primary care: first choice and restrictive prescribing are two different traits. Qual Saf Health Care. 2007;16(2):105-9.

77.	Vichare A, Eads N, Punglia R, Potters L. American Society for Radiation Oncology's Performance Assessment for the Advancement of Radiation Oncology Treatment: A practical approach for informing practice improvement. Pract Radiat Oncol. 2013;3(2):e37-43.

78.	Wang Y, Li Z, Zhao X, Wang C, Wang X, Wang D, et al. Effect of a Multifaceted Quality Improvement Intervention on Hospital Personnel Adherence to Performance Measures in Patients With Acute Ischemic Stroke in China: A Randomized Clinical Trial. Jama. 2018;320(3):245-54.

79.	Wang C, Su S, Li X, Li J, Bao X, Liu M. Identifying Performance Outliers for Stroke Care Based on Composite Score of Process Indicators: an Observational Study in China. J Gen Intern Med. 2020;35(9):2621-8.

80.	Weng W, Hess BJ, Lynn LA, Lipner RS. Assessing the Quality of Osteoporosis Care in Practice. J Gen Intern Med. 2015;30(11):1681-7.

81.	Willis CD, Stoelwinder JU, Lecky FE, Woodford M, Jenks T, Bouamra O, et al. Applying composite performance measures to trauma care. Journal of Trauma - Injury, Infection and Critical Care. 2010;69(2):256-62.

82.	Wu Y, Li S, Patel A, Li X, Du X, Wu T, et al. Effect of a Quality of Care Improvement Initiative in Patients With Acute Coronary Syndrome in Resource-Constrained Hospitals in China: A Randomized Clinical Trial. JAMA Cardiol. 2019;4(5):418-27.

83.	Zeitlin J, Manktelow BN, Piedvache A, Cuttini M, Boyle E, van Heijst A, et al. Use of evidence based practices to improve survival without severe morbidity for very preterm infants: results from the EPICE population based cohort. Bmj. 2016;354:i2976.

84.	Zerillo JA, Pham TH, Kadlubek P, Severson JA, Mackler E, Jacobson JO, et al. Administration of oral chemotherapy: results from three rounds of the quality oncology practice initiative. J Oncol Pract. 2015;11(2):e255-62.

85.	Zhang X, Li Z, Zhao X, Xian Y, Liu L, Wang C, et al. Relationship between hospital performance measures and outcomes in patients with acute ischaemic stroke: a prospective cohort study. BMJ Open. 2018;8(7):e020467.

86.	Zuckerman B, Stevens GD, Inkelas M, Halfon N. Prevalence and correlates of high-quality basic pediatric preventive care. Pediatrics. 2004;114(6):1522-9.

87.	Alasfoor D. Exploring access to primary health care among diabetic patients In Oman: University of Oxford; 2020.

88.	Ayton D, Soh SE, Morello R, Ahern S, Earnest A, Brennan A, et al. Development of a percutaneous coronary intervention patient level composite measure for a clinical quality registry. BMC Health Serv Res. 2020;20(1):44.

89.	Badhwar V, Rankin JS, He X, Jacobs JP, Gammie JS, Furnary AP, et al. The Society of Thoracic Surgeons Mitral Repair/Replacement Composite Score: A Report of The Society of Thoracic Surgeons Quality Measurement Task Force. Ann Thorac Surg. 2016;101(6):2265-71.

90.	Barbaree HE, Perlman C, Ham E, Brown GP, Hirdes JP. Validation of a composite outcome measure for inpatient psychiatry using scales from the interRAI-MH. Front Psychiatry. 2025;16:1486734.

91.	Beane JD, Borrebach JD, Zureikat AH, Kilbane EM, Thompson VM, Pitt HA. Optimal Pancreatic Surgery: Are We Making Progress in North America? Ann Surg. 2021;274(4):e355-e63.

92.	Boyle DK, Jayawardhana A, Burman ME, Dunton NE, Staggs VS, Bergquist-Beringer S, et al. A pressure ulcer and fall rate quality composite index for acute care units: A measure development study. Int J Nurs Stud. 2016;63:73-81.

93.	Bozic KJ, Grosso LM, Lin Z, Parzynski CS, Suter LG, Krumholz HM, et al. Variation in hospital-level risk-standardized complication rates following elective primary total hip and knee arthroplasty. J Bone Joint Surg Am. 2014;96(8):640-7.

94.	Broderick SR, Grau-Sepulveda M, Kosinski AS, Kurlansky PA, Shahian DM, Jacobs JP, et al. The Society of Thoracic Surgeons Composite Score Rating for Pulmonary Resection for Lung Cancer. Ann Thorac Surg. 2020;109(3):848-55.

95.	Carey K, Zhao S, Snow AL, Hartmann CW. The relationship between nursing home quality and costs: Evidence from the VA. PLoS One. 2018;13(9):e0203764.

96.	Cartland J, Green M, Kamm D, Halfer D, Brisk MA, Wheeler D. Measuring psychological safety and local learning to enable high reliability organisational change. BMJ Open Qual. 2022;11(4).

97.	Caruso G, Nasioudis D, McGree M, Fought A, Fumagalli D, Reynolds E, et al. ACHIEVING A TEXTBOOK ONCOLOGIC OUTCOME AFTER ENDOMETRIAL CANCER SURGERY IS ASSOCIATED WITH IMPROVED LONG-TERM SURVIVAL. International Journal of Gynecological Cancer. 2024;34:A145-A6.

98.	Comart J, Mahler A, Schreiber R, Rockett C, Jones RN, Morris JN. Palliative care for long-term care residents: effect on clinical outcomes. Gerontologist. 2013;53(5):874-80.

99.	Danilyants N, MacKoul P, van der Does L, Haworth L, Baxi R. A value-based evaluation of minimally invasive hysterectomy approaches. Gynecological Surgery. 2019;16(1).

100.	Desai ND, O'Brien SM, Cohen DJ, Carroll J, Vemulapalli S, Arnold SV, et al. Composite Metric for Benchmarking Site Performance in Transcatheter Aortic Valve Replacement: Results From the STS/ACC TVT Registry. Circulation. 2021;144(3):186-94.

101.	Dimick JB, Staiger DO, Hall BL, Ko CY, Birkmeyer JD. Composite measures for profiling hospitals on surgical morbidity. Ann Surg. 2013;257(1):67-72.

102.	Dooley J, Jardine J, Ibrahim B, Mongru R, Pradhan F, Wolstenholme D, et al. A positive deviant approach to examining the impact of Covid-19 on ethnic inequalities in maternal and neonatal outcomes. Sexual and Reproductive Healthcare. 2024;40.

103.	Dunham AL, Ramirez LD, Vang CA, Linebarger JH, Landercasper J. Profiling Surgeon Performance for Breast Cancer Lumpectomy by Composite Measurement of Reoperations, Cosmetic Outcomes, and Patient Preferences. Ann Surg Oncol. 2018;25(7):1943-52.

104.	Eklom B, Tracy S, Callander E. An exploration of potential output measures to assess efficiency and productivity for labour and birth in Australia. BMC Pregnancy Childbirth. 2021;21(1):703.

105.	Glance LG, Hasley S, Glantz JC, Stevens TP, Faden E, Kreso MA, et al. Measuring Childbirth Outcomes Using Administrative and Birth Certificate Data. Anesthesiology. 2019;131(2):238-53.

106.	Gorgemans S, Comendeiro-Maaløe M, Ridao-López M, Bernal-Delgado E. Quality and technical efficiency do not evolve hand in hand in Spanish hospitals: Observational study with administrative data. PLoS One. 2018;13(8):e0201466.

107.	Hardt J, Doyon F, Klinger C, Buhr HJ, Post S. MTL, a composite measure for objectively profiling hospitals on surgical performance in colorectal cancer surgery. Int J Colorectal Dis. 2019;34(5):889-98.

108.	Hartman N, Messana JM, Kang J, Naik AS, Shearon TH, He K. COMPOSITE SCORES FOR TRANSPLANT CENTER EVALUATION: A NEW INDIVIDUALIZED EMPIRICAL NULL METHOD. Ann Appl Stat. 2024;18(1):729-48.

109.	Hermer L, Cornelison L, Kaup ML, Poey JL, Drake PN, Stone RI, et al. Person-Centered Care as Facilitated by Kansas' PEAK 2.0 Medicaid Pay-for-Performance Program and Nursing Home Resident Clinical Outcomes. Innov Aging. 2018;2(3):igy033.

110.	Hofstede SN, Ceyisakar IE, Lingsma HF, Kringos DS, Marang-van de Mheen PJ. Ranking hospitals: do we gain reliability by using composite rather than individual indicators? BMJ Qual Saf. 2019;28(2):94-102.

111.	Hollenbeck B, Hoffman MA, Tromanhauser SG. High-Volume Arthroplasty Centers Demonstrate Higher Composite Quality Scores and Enhanced Value: Perspective on Higher-Volume Hospitals Performing Arthroplasty from 2001 to 2011. J Bone Joint Surg Am. 2020;102(5):362-7.

112.	Hyer JM, Diaz A, Pawlik TM. The win ratio: A novel approach to define and analyze postoperative composite outcomes to reflect patient and clinician priorities. Surgery (United States). 2022;172(5):1484-9.

113.	Kaempf JW, Zupancic JA, Wang L, Grunkemeier GL. A risk-adjusted, composite outcomes score and resource utilization metrics for very low-birth-weight infants. JAMA Pediatr. 2015;169(5):459-65.

114.	Karthaus EG, Lijftogt N, Busweiler LAD, Elsman BHP, Wouters M, Vahl AC, et al. Textbook Outcome: A Composite Measure for Quality of Elective Aneurysm Surgery. Ann Surg. 2017;266(5):898-904.

115.	Keller DS, Chien H-L, Hashemi L, Senagore AJ, Delaney CP. The HARM score: a novel, easy measure to evaluate quality and outcomes in colorectal surgery. Annals of surgery. 2014;259(6):1119-25.

116.	Kozower BD, O'Brien SM, Kosinski AS, Magee MJ, Dokholyan R, Jacobs JP, et al. The Society of Thoracic Surgeons Composite Score for Rating Program Performance for Lobectomy for Lung Cancer. Ann Thorac Surg. 2016;101(4):1379-86; discussion 86-7.

117.	Kuhrij LS, Karthaus EG, Vahl AC, Willems MCM, Elshof JW, de Borst GJ. A Composite Measure for Quality of Care in Patients with Symptomatic Carotid Stenosis Using Textbook Outcome. Eur J Vasc Endovasc Surg. 2020;60(4):502-8.

118.	Lain SJ, Algert CS, Nassar N, Bowen JR, Roberts CL. Incidence of severe adverse neonatal outcomes: use of a composite indicator in a population cohort. Matern Child Health J. 2012;16(3):600-8.

119.	Lawson KA, Daignault K, Abouassaly R, Khanna A, Martin L, Goldenberg M, et al. Hospital-level Effects Contribute to Variations in Prostate Cancer Quality of Care. Eur Urol Oncol. 2021;4(3):494-7.

120.	Li P, Schneider JE, Ward MM. Effect of critical access hospital conversion on patient safety. Health Serv Res. 2007;42(6 Pt 1):2089-108; discussion 294-323.

121.	Li T, Curtin-Bowen M, Pullman A, Lipsitz S, Syrowatka A, Sainlaire M, et al. Testing of a Risk-Standardized Major Bleeding and Venous Thromboembolism Electronic Clinical Quality Measure for Elective Total Hip and/or Knee Arthroplasties. AMIA Annu Symp Proc. 2021;2021:736-43.

122.	Lingsma HF, Bottle A, Middleton S, Kievit J, Steyerberg EW, Marang-van de Mheen PJ. Evaluation of hospital outcomes: the relation between length-of-stay, readmission, and mortality in a large international administrative database. BMC Health Serv Res. 2018;18(1):116.

123.	Mann S, Pratt S, Gluck P, Nielsen P, Risser D, Greenberg P, et al. Assessing quality in obstetrical care: development of standardized measures. The Joint Commission Journal on Quality and Patient Safety. 2006;32(9):497-505.

124.	Mansfield JG, Caplan RA, Campos JS, Dreis DF, Furman C. Using a quantitative risk register to promote learning from a patient safety reporting system. Jt Comm J Qual Patient Saf. 2015;41(2):76-86.

125.	Merath K, Chen Q, Bagante F, Beal E, Akgul O, Dillhoff M, et al. Textbook Outcomes Among Medicare Patients Undergoing Hepatopancreatic Surgery. Ann Surg. 2020;271(6):1116-23.

126.	Milliren CE, Bailey G, Graham DA, Ozonoff A. Relationships Between Pediatric Safety Indicators Across a National Sample of Pediatric Hospitals: Dispelling the Myth of the "Safest" Hospital. J Patient Saf. 2022;18(4):e741-e6.

127.	Nair M, Kurinczuk JJ, Knight M. Establishing a National Maternal Morbidity Outcome Indicator in England: A Population-Based Study Using Routine Hospital Data. PLoS One. 2016;11(4):e0153370.

128.	Neary C, O'Brien L, McCormack E, Kelly M, Bolger J, McEntee G, et al. Defining a textbook outcome for the resection of colorectal liver metastases. Journal of Surgical Oncology. 2023;127(4):616-24.

129.	Numan RC. Monitoring and improving care in thoracic surgery: Universiteit van Amsterdam [Host]; 2016.

130.	Pasquali SK, Shahian DM, O'Brien SM, Jacobs ML, Gaynor JW, Romano JC, et al. Development of a Congenital Heart Surgery Composite Quality Metric: Part 1-Conceptual Framework. Ann Thorac Surg. 2019;107(2):583-9.

131.	Patterson PD, Weaver MD, Frank RC, Warner CW, Martin-Gill C, Guyette FX, et al. Association between poor sleep, fatigue, and safety outcomes in emergency medical services providers. Prehospital Emergency Care. 2012;16(1):86-97.

132.	Pitocco C, Sexton TR. Using Data Analytics to Improve Nursing Home Quality. Qual Manag Health Care. 2022.

133.	Poelemeijer YQM, Marang-van de Mheen PJ, Wouters M, Nienhuijs SW, Liem RSL. Textbook Outcome: an Ordered Composite Measure for Quality of Bariatric Surgery. Obes Surg. 2019;29(4):1287-94.

134.	Ramani S, Halpern TA, Akerman M, Ananth CV, Vintzileos AM. A new index for obstetrics safety and quality of care: integrating cesarean delivery rates with maternal and neonatal outcomes. Am J Obstet Gynecol. 2022;226(4):556.e1-.e9.

135.	Rankin JS, Badhwar V, He X, Jacobs JP, Gammie JS, Furnary AP, et al. The Society of Thoracic Surgeons Mitral Valve Repair/Replacement Plus Coronary Artery Bypass Grafting Composite Score: A Report of The Society of Thoracic Surgeons Quality Measurement Task Force. Ann Thorac Surg. 2017;103(5):1475-81.

136.	Reponen E, Tuominen H, Hernesniemi J, Korja M. Patient-Reported Outcomes in Elective Cranial Neurosurgery. World Neurosurg. 2015;84(6):1845-51.

137.	Rosen AK, Loveland SA, Romano PS, Itani KM, Silber JH, Even-Shoshan OO, et al. Effects of resident duty hour reform on surgical and procedural patient safety indicators among hospitalized Veterans Health Administration and Medicare patients. Med Care. 2009;47(7):723-31.

138.	Rosen AK, Chen Q, Borzecki AM, Shin M, Itani KM, Shwartz M. Using estimated true safety event rates versus flagged safety event rates: does it change hospital profiling and payment? Health Serv Res. 2014;49(5):1426-45.

139.	Salet N, Bremmer RH, Verhagen M, Ekkelenkamp VE, Hansen BE, de Jonge PJF, et al. Is Textbook Outcome a valuable composite measure for short-term outcomes of gastrointestinal treatments in the Netherlands using hospital information system data? A retrospective cohort study. BMJ Open. 2018;8(2):e019405.

140.	Sammer C, Hauck LD, Jones C, Zaiback-Aldinger J, Li M, Classen D. Examining the Relationship of an All-Cause Harm Patient Safety Measure and Critical Performance Measures at the Frontline of Care. J Patient Saf. 2020;16(1):110-6.

141.	Seder CW, Raymond DP, Wright CD, Gaissert HA, Chang AC, Clinton S, et al. The Society of Thoracic Surgeons General Thoracic Surgery Database 2017 Update on Outcomes and Quality. Ann Thorac Surg. 2017;103(5):1378-83.

142.	Shahian DM, He X, Jacobs JP, Rankin JS, Welke KF, Filardo G, et al. The Society of Thoracic Surgeons Isolated Aortic Valve Replacement (AVR) Composite Score: a report of the STS Quality Measurement Task Force. Ann Thorac Surg. 2012;94(6):2166-71.

143.	Shahian DM, He X, Jacobs JP, Rankin JS, Welke KF, Edwards FH, et al. The STS AVR+CABG composite score: a report of the STS Quality Measurement Task Force. Ann Thorac Surg. 2014;97(5):1604-9.

144.	Shahian DM, He X, Jacobs JP, Kurlansky PA, Badhwar V, Cleveland JC, Jr., et al. The Society of Thoracic Surgeons Composite Measure of Individual Surgeon Performance for Adult Cardiac Surgery: A Report of The Society of Thoracic Surgeons Quality Measurement Task Force. Ann Thorac Surg. 2015;100(4):1315-24; discussion 24-5.

145.	Shahian DM, Badhwar V, Kurlansky PA, Bowdish ME, Lobdell KW, Furnary AP, et al. The STS Participant-Level, Multiprocedural Composite Measure for Adult Cardiac Surgery. Ann Thorac Surg. 2022;114(2):467-75.

146.	Shwartz M, Peköz EA, Burgess JF, Jr., Christiansen CL, Rosen AK, Berlowitz D. A probability metric for identifying high-performing facilities: an application for pay-for-performance programs. Med Care. 2014;52(12):1030-6.

147.	Smith PK, Amster A. An Organization-Specific and Modifiable Inpatient Safety Composite Measure. Jt Comm J Qual Patient Saf. 2019;45(4):304-14.

148.	Smith K, McAvoy J, Six JD, Goss L. A Composite Score Reflecting an Initiative to Reduce Harm, Protect Patients, and Promote Safety. J Healthc Qual. 2025;47(1).

149.	Straneo M, Hanson C, van den Akker T, Afolabi BB, Asefa A, Delamou A, et al. Inequalities in use of hospitals for childbirth among rural women in sub-Saharan Africa: a comparative analysis of 18 countries using Demographic and Health Survey data. BMJ Glob Health. 2024;9(1).

150.	Sullivan JL, Shwartz M, Burgess JF, Jr., Peköz EA, Christiansen CL, Gerena-Melia M, et al. Person-centered care practices and quality in Department of Veterans Affairs nursing homes: is there a relationship? Med Care. 2013;51(2):165-71.

151.	Ten Berge MG, Beck N, Steup WH, Verhagen A, van Brakel TJ, Schreurs WH, et al. Textbook outcome as a composite outcome measure in non-small-cell lung cancer surgery. Eur J Cardiothorac Surg. 2021;59(1):92-9.

152.	Society of Thoracic Surgeons General Thoracic Surgery Database Task F. The Society of Thoracic Surgeons Composite Score for Evaluating Esophagectomy for Esophageal Cancer. Ann Thorac Surg. 2017;103(5):1661-7.

153.	van der Heide MFJ, de Jel DVC, Hoeijmakers F, Hoebers FJP, de Boer JP, Hamming-Vrieze O, et al. Defining High-Quality Integrated Head and Neck Cancer Care Through a Composite Outcome Measure: Textbook Outcome. Laryngoscope. 2022;132(1):78-87.

154.	Warps AK, Detering R, Tollenaar R, Tanis PJ, Dekker JWT. Textbook outcome after rectal cancer surgery as a composite measure for quality of care: A population-based study. Eur J Surg Oncol. 2021;47(11):2821-9.

155.	Watt H, Harris M, Noyes J, Whitaker R, Hoare Z, Edwards RT, et al. Development of a composite outcome score for a complex intervention - measuring the impact of Community Health Workers. Trials. 2015;16:107.

156.	Weaver MD, Wang HE, Fairbanks RJ, Patterson D. The association between EMS workplace safety culture and safety outcomes. Prehosp Emerg Care. 2012;16(1):43-52.

157.	Wiseman JT, Cloyd JM, Shelby R, Suarez-Kelly L, Ethun CG, Tran TB, et al. Analysis of textbook outcomes among patients undergoing resection of retroperitoneal sarcoma: A multi-institutional of the u.s. sarcoma collaborative. Annals of Surgical Oncology. 2020;27:S52.

158.	Wiseman JT, Abdel-Misih S, Beal EW, Zaidi MY, Staley CA, Grotz T, et al. A multi-institutional analysis of Textbook Outcomes among patients undergoing cytoreductive surgery for peritoneal surface malignancies. Surg Oncol. 2021;37:101492.

159.	Zaslavsky AM, Shaul JA, Zaborski LB, Cioffi MJ, Cleary PD. Combining health plan performance indicators into simpler composite measures. Health Care Financ Rev. 2002;23(4):101-15.

160.	Zogg CK, Cooper Z, Peduzzi P, Falvey JR, Castillo-Angeles M, Kodadek LM, et al. Changes in Older Adult Trauma Quality When Evaluated Using Longer-Term Outcomes vs In-Hospital Mortality. JAMA Surg. 2023;158(12):e234856.

161.	Aaronson DS, Bardach NS, Lin GA, Chattopadhyay A, Goldman LE, Dudley RA. Prediction of hospital acute myocardial infarction and heart failure 30-day mortality rates using publicly reported performance measures. J Healthc Qual. 2013;35(2):15-23.

162.	Agniel D, Martino SC, Burkhart Q, Hambarsoomian K, Orr N, Beckett MK, et al. Incentivizing Excellent Care to At-Risk Groups with a Health Equity Summary Score. J Gen Intern Med. 2021;36(7):1847-57.

163.	Allorant A, Parrish C, Desforges G, Robin E, Honore JG, Puttkammer N. Closing the gap in implementation of HIV clinical guidelines in a low resource setting using electronic medical records. BMC Health Serv Res. 2020;20(1):804.

164.	Amoah AO, Amirfar S, Sebek K, Silfen SL, Singer J, Wang JJ. Developing Composite Quality Measures for EHR-Enabled Primary Care Practices in New York City. J Med Pract Manage. 2015;30(4):231-9.

165.	Anthony MK, Brennan PF, O'Brien R, Suwannaroop N. Measurement of nursing practice models using multiattribute utility theory: relationship to patient and organizational outcomes. Qual Manag Health Care. 2004;13(1):40-52.

166.	Aoki A, Mochida K, Kuramata M, Sadamori T, Sapalalo P, Tchicondingosse L, et al. Association between the quality of care and continuous maternal and child health service utilisation in Angola: Longitudinal data analysis. J Glob Health. 2023;13:04073.

167.	Aragon L, Schieman K, Cure L. Incorporating the six aims for quality in the analysis of trauma care. Health Syst (Basingstoke). 2022;11(2):98-108.

168.	Ashby J, Juarez DT, Berthiaume J, Sibley P, Chung RS. The relationship of hospital quality and cost per case in Hawaii. Inquiry. 2012;49(1):65-74.

169.	Austin JM, D'Andrea G, Birkmeyer JD, Leape LL, Milstein A, Pronovost PJ, et al. Safety in numbers: the development of Leapfrog's composite patient safety score for U.S. hospitals. J Patient Saf. 2014;10(1):64-71.

170.	Bilimoria KY, Bentrem DJ, Lillemoe KD, Talamonti MS, Ko CY. Assessment of pancreatic cancer care in the United States based on formally developed quality indicators. J Natl Cancer Inst. 2009;101(12):848-59.

171.	Bilimoria KY, Raval MV, Bentrem DJ, Wayne JD, Balch CM, Ko CY. National assessment of melanoma care using formally developed quality indicators. J Clin Oncol. 2009;27(32):5445-51.

172.	Brewer BB, Verran JA. Measuring nursing unit environments with four composite measure. Nurs Econ. 2013;31(5):241-9.

173.	Brown EG, Bateni SB, Burgess D, Li CS, Bold RJ. Interhospital Variability in Quality Outcomes of Pancreatic Surgery. J Surg Res. 2019;235:453-8.

174.	Brunelli A, Berrisford RG, Rocco G, Varela G. The European Thoracic Database project: composite performance score to measure quality of care after major lung resection. European journal of cardio-thoracic surgery. 2009;35(5):769-74.

175.	Brunelli A, Rocco G, Van Raemdonck D, Varela G, Dahan M. Lessons learned from the European thoracic surgery database: the Composite Performance Score. European Journal of Surgical Oncology (EJSO). 2010;36:S93-S9.

176.	Bruzzi S, Ivaldi E, Santagata M. Measuring Regional Performance in the Italian NHS: Are Disparities Decreasing? Soc Indic Res. 2022;159(3):1057-84.

177.	Cabrera-Barona P, Blaschke T, Kienberger S. Explaining Accessibility and Satisfaction Related to Healthcare: A Mixed-Methods Approach. Soc Indic Res. 2017;133(2):719-39.

178.	Calciolari S, Ilinca S. Unraveling care integration: Assessing its dimensions and antecedents in the Italian Health System. Health Policy. 2016;120(1):129-38.

179.	Calderon-Margalit R, Cohen-Dadi M, Opas D, Jaffe DH, Levine J, Ben-Yehuda A, et al. Trends in the performance of quality indicators for diabetes care in the community and in diabetes-related health status: an Israeli ecological study. Isr J Health Policy Res. 2018;7(1):10.

180.	Carey K, Luo Q, Dor A. Quality and Cost in Community Health Centers. Med Care. 2021;59(9):824-8.

181.	Chen T-T, Lai M-S, Lin IC, Chung K-P. Exploring and Comparing the Characteristics of Nonlatent and Latent Composite Scores: Implications for Pay-for-Performance Incentive Design. Medical Decision Making. 2012;32(1):132-44.

182.	Chen LM, Staiger DO, Birkmeyer JD, Ryan AM, Zhang W, Dimick JB. Composite quality measures for common inpatient medical conditions. Med Care. 2013;51(9):832-7.

183.	Choi SE, Kalenderian E, Sharon‐Lise N. Measuring the quality of dental care among privately insured children in the United States. Health Services Research. 2022;57(1):137-44.

184.	Damberg CL, Shortell SM, Raube K, Gillies RR, Rittenhouse D, McCurdy RK, et al. Relationship between quality improvement processes and clinical performance. Am J Manag Care. 2010;16(8):601-6.

185.	Katz D, A. , Wu C, Jaske E, Stewart G, L. , Mohr D, C. . Care Practices to Promote Patient Engagement in VA Primary Care: Factors Associated With High Performance. The Annals of Family Medicine. 2020;18(5):397.

186.	Dehanne F, Pirson M, Cuvelier E, Bielen F, Leclercq P, Libert B, et al. An inter-hospital performance assessment model for evaluating hospitals performing hip arthroplasty. Healthcare Analytics. 2024;6.

187.	de Miranda Costa MM, Santana HT, Saturno Hernandez PJ, Carvalho AA, da Silva Gama ZA. Results of a national system-wide quality improvement initiative for the implementation of evidence-based infection prevention practices in Brazilian hospitals. J Hosp Infect. 2020;105(1):24-34.

188.	de Oliveira Valentino TC, Paiva CE, Hui D, de Oliveira MA, Ribeiro Paiva BS. Impact of Palliative Care on Quality of End-of-Life Care Among Brazilian Patients With Advanced Cancers. J Pain Symptom Manage. 2020;59(1):39-48.

189.	Dhillon PK, Hallowell BD, Agrawal S, Ghosh A, Yadav A, Van Dyne E, et al. Is India's public health care system prepared for cervical cancer screening?: Evaluating facility readiness from the fourth round of the District Level Household and Facility Survey (DLHS-4). Preventive Medicine. 2020;138.

190.	Dimick JB, Staiger DO, Baser O, Birkmeyer JD. Composite measures for predicting surgical mortality in the hospital. Health Aff (Millwood). 2009;28(4):1189-98.

191.	Dimick JB, Staiger DO, Osborne NH, Nicholas LH, Birkmeyer JD. Composite measures for rating hospital quality with major surgery. Health Serv Res. 2012;47(5):1861-79.

192.	Dimick JB, Birkmeyer NJ, Finks JF, Share DA, English WJ, Carlin AM, et al. Composite measures for profiling hospitals on bariatric surgery performance. JAMA Surg. 2014;149(1):10-6.

193.	Dong Y, Yang X, Fang P, Pan Z, Luo Z. Have the Pilot County Hospitals' Service Capability Been Improved Since the Healthcare Reform? An Analysis of 370 Hospitals in China. Iran J Public Health. 2019;48(3):474-83.

194.	Duggal B, Duggal M, Panch A, Chourase M, Gedam P, Singh P, et al. Using a national level cross-sectional study to develop a Hospital Preparedness Index (HOSPI) for Covid-19 management: A case study from India. PLoS One. 2022;17(7):e0269842.

195.	Dusheiko M, Gravelle H, Martin S, Smith PC. Quality of Disease Management and Risk of Mortality in English Primary Care Practices. Health Serv Res. 2015;50(5):1452-71.

196.	Ekouevi DK, Stringer E, Coetzee D, Tih P, Creek T, Stinson K, et al. Health facility characteristics and their relationship to coverage of PMTCT of HIV services across four African countries: the PEARL study. PLoS One. 2012;7(1):e29823.

197.	Ferreira DC, Nunes AM, Marques RC. Operational efficiency vs clinical safety, care appropriateness, timeliness, and access to health care. Journal of Productivity Analysis. 2020;53(3):355-75.

198.	Fieldston ES, Zaoutis LB, Agosto PM, Guo A, Jonas JA, Tsarouhas N. Measuring patient flow in a children's hospital using a scorecard with composite measurement. Journal of Hospital Medicine. 2014;9(7):463-8.

199.	Fong J, Marsh GM, Stokan LA, Weilian S, Vinson C, Ruhl L. Hospital quality performance report: an application of composite scoring. Am J Med Qual. 2008;23(4):287-95.

200.	Fruhauf T, Zimmerman L, Kibira SPS, Makumbi F, Gichangi P, Shiferaw S, et al. Measuring family planning quality and its link with contraceptive use in public facilities in Burkina Faso, Ethiopia, Kenya and Uganda. Health Policy Plan. 2018;33(7):828-39.

201.	Gage AD, Leslie HH, Bitton A, Jerome JG, Thermidor R, Joseph JP, et al. Assessing the quality of primary care in Haiti. Bull World Health Organ. 2017;95(3):182-90.

202.	Gage AD, Leslie HH, Bitton A, Jerome JG, Joseph JP, Thermidor R, et al. Does quality influence utilization of primary health care? Evidence from Haiti. Global Health. 2018;14(1):59.

203.	Gajewski BJ, Dunton N. Identifying individual changes in performance with composite quality indicators while accounting for regression to the mean. Med Decis Making. 2013;33(3):396-406.

204.	Garrido-Cumbrera M, Almenara-Barrios J, López-Lara E, Peralta-Sáez JL, García-Gutierrez JC, Salvador-Carulla L. Development and spatial representation of synthetic indexes of outpatient mental health care in Andalusia (Spain). Epidemiol Psichiatr Soc. 2008;17(3):192-200.

205.	Getachew T, Abebe SM, Yitayal M, Persson L, Berhanu D. Assessing the quality of care in sick child services at health facilities in Ethiopia. BMC Health Serv Res. 2020;20(1):574.

206.	Goh R, Hillier S, Kelly TL, Worley A, Dixon K, Kurunawai C, et al. Implementation of the South Australian Regional Telestroke service is associated with improved care quality and lower stroke mortality: A retrospective cohort study. Australian Journal of Rural Health. 2023;31(5):878-85.

207.	Hamad DM, Subacius H, Thomas A, Guttman MP, Tillmann BW, Jerath A, et al. A multidimensional approach to identifying high-performing trauma centers across the United States. J Trauma Acute Care Surg. 2024;97(1):125-33.

208.	Hess BJ, Weng W, Lynn LA, Holmboe ES, Lipner RS. Setting a fair performance standard for physicians' quality of patient care. J Gen Intern Med. 2011;26(5):467-73.

209.	Hicks LS, O'Malley AJ, Lieu TA, Keegan T, McNeil BJ, Guadagnoli E, et al. Impact of health disparities collaboratives on racial/ethnic and insurance disparities in US community health centers. Arch Intern Med. 2010;170(3):279-86.

210.	Holbrook A, Thabane L, Keshavjee K, Dolovich L, Bernstein B, Chan D, et al. Individualized electronic decision support and reminders to improve diabetes care in the community: COMPETE II randomized trial. Cmaj. 2009;181(1-2):37-44.

211.	Holmboe ES, Arnold GK, Weng W, Lipner R. Current yardsticks may be inadequate for measuring quality improvements from the medical home. Health Aff (Millwood). 2010;29(5):859-66.

212.	Holmboe ES, Weng W, Arnold GK, Kaplan SH, Normand SL, Greenfield S, et al. The comprehensive care project: measuring physician performance in ambulatory practice. Health Serv Res. 2010;45(6 Pt 2):1912-33.

213.	Horberg MA, Certa JM, Rubenstein KB, Hurley LB, Satre DD, Kadlecik PM, et al. Beyond the HIV care continuum and viral suppression: Broadening the scope of quality metrics for total HIV patient care. AIDS Patient Care and STDs. 2020;34(11):461-9.

214.	Horwood C, Haskins L, Phakathi S, McKerrow N. A health systems strengthening intervention to improve quality of care for sick and small newborn infants: results from an evaluation in district hospitals in KwaZulu-Natal, South Africa. BMC Pediatr. 2019;19(1):29.

215.	Hota B, Webb T, Chatrathi A, McAninch E, Lateef O. Disagreement Between Hospital Rating Systems: Measuring the Correlation of Multiple Benchmarks and Developing a Quality Composite Rank. Am J Med Qual. 2020;35(3):222-30.

216.	Iyengar B, Rahul S, Mossner B, James M, Sekhri B, Mullard M, et al. A New Composite Measure for Assessing Surgical Performance. Michigan Journal of Medicine. 2019;4(1).

217.	Jain AK. A New Composite Index to Measure National-Level Quality of Family Planning Programs. Int Perspect Sex Reprod Health. 2018;44(2):63-72.

218.	Kadoić N, Šimić D, Mesarić J, Begičević Ređep N. Measuring Quality of Public Hospitals in Croatia Using a Multi-Criteria Approach. Int J Environ Res Public Health. 2021;18(19).

219.	Kane RL, Arling G, Mueller C, Held R, Cooke V. A quality-based payment strategy for nursing home care in Minnesota. Gerontologist. 2007;47(1):108-15.

220.	Kaplan SH, Griffith JL, Price LL, Pawlson LG, Greenfield S. Improving the reliability of physician performance assessment: identifying the "physician effect" on quality and creating composite measures. Med Care. 2009;47(4):378-87.

221.	Katz A, Herpai N, Smith G, Aubrey-Bassler K, Breton M, Boivin A, et al. Alignment of Canadian Primary Care With the Patient Medical Home Model: A QUALICO-PC Study. Ann Fam Med. 2017;15(3):230-6.

222.	Kumar H, Khanna R, Alwadhi V, Bhat AA, Neogi SB, Choudhry P, et al. Catalytic Support for Improving Clinical Care in Special Newborn Care Units (SNCU) Through Composite SNCU Quality of Care Index (SQCI). Indian Pediatr. 2021;58(4):338-44.

223.	Lalloué B, Jiang S, Girault A, Ferrua M, Loirat P, Minvielle E. Evaluation of the effects of the French pay-for-performance program—IFAQ pilot study. International Journal for quality in Health care. 2017;29(6):833-7.

224.	Landon BE, Hicks LS, O'Malley AJ, Lieu TA, Keegan T, McNeil BJ, et al. Improving the management of chronic disease at community health centers. N Engl J Med. 2007;356(9):921-34.

225.	Landrum MB, Bronskill SE, Normand S-LT. Analytic Methods for Constructing Cross-Sectional Profiles of Health Care Providers. Health Services & Outcomes Research Methodology. 2000;1(1):23-47.

226.	Lawson KA, Saarela O, Abouassaly R, Kim SP, Breau RH, Finelli A. The Impact of Quality Variations on Patients Undergoing Surgery for Renal Cell Carcinoma: A National Cancer Database Study. Eur Urol. 2017;72(3):379-86.

227.	Leegwater A, Wong W, Avila C. A concise, health service coverage index for monitoring progress towards universal health coverage. BMC Health Serv Res. 2015;15:230.

228.	Lemke S, Boden MT, Kearney LK, Krahn DD, Neuman MJ, Schmidt EM, et al. Measurement-based management of mental health quality and access in VHA: SAIL mental health domain. Psychological services. 2017;14(1):1.

229.	Levine DA, Funkhouser EM, Houston TK, Gerald JK, Johnson-Roe N, Allison JJ, et al. Improving care after myocardial infarction using a 2-year internet-delivered intervention: the Department of Veterans Affairs myocardial infarction-plus cluster-randomized trial. Arch Intern Med. 2011;171(21):1910-7.

230.	Lied TR, Malsbary R, Eisenberg C, Ranck J. Combining HEDIS indicators: a new approach to measuring plan performance. Health Care Financ Rev. 2002;23(4):117-29.

231.	Lipner RS, Weng W, Caverzagie KJ, Hess BJ. Physician performance assessment: prevention of cardiovascular disease. Adv Health Sci Educ Theory Pract. 2013;18(5):1029-45.

232.	Liu K, Jain S, Shi J. Physician performance assessment using a composite quality index. Stat Med. 2013;32(15):2661-80.

233.	Portela MCAS, Camanho AS, Almeida DQ, Lopes L, Silva SN, Castro R. Benchmarking hospitals through a web based platform. Benchmarking. 2016;23(3):722-39.

234.	Matos R, Ferreira D, Pedro MI. Economic Analysis of Portuguese Public Hospitals Through the Construction of Quality, Efficiency, Access, and Financial Related Composite Indicators. Soc Indic Res. 2021;157(1):361-92.

235.	Mays GP, McHugh MC, Shim K, Perry N, Halverson PK, Lenaway D, et al. Identifying dimensions of performance in local public health systems: results from the National Public Health Performance Standards Program. J Public Health Manag Pract. 2004;10(3):193-203.

236.	Megwalu UC, Ma Y, Hernandez-Boussard T, Divi V, Gomez SL. The Impact of Hospital Quality on Thyroid Cancer Survival. Otolaryngol Head Neck Surg. 2020;162(3):269-76.

237.	Modi D, Dholakia N, Gopalan R, Venkatraman S, Dave K, Shah S, et al. mHealth intervention "ImTeCHO" to improve delivery of maternal, neonatal, and child care services-A cluster-randomized trial in tribal areas of Gujarat, India. PLoS Med. 2019;16(10):e1002939.

238.	Monsees E, Goldman J, Vogelsmeier A, Popejoy L. Nurses as antimicrobial stewards: Recognition, confidence, and organizational factors across nine hospitals. Am J Infect Control. 2020;48(3):239-45.

239.	Mulder C, Rayner J. High and Sustained Participation in a Multi-year Voluntary Performance Measurement Initiative Among Primary Care Teams. International journal of health policy and management. 2022;11(4):514-20.

240.	Nelson KM, Helfrich C, Sun H, Hebert PL, Liu CF, Dolan E, et al. Implementation of the patient-centered medical home in the Veterans Health Administration: associations with patient satisfaction, quality of care, staff burnout, and hospital and emergency department use. JAMA Intern Med. 2014;174(8):1350-8.

241.	Normand ST, Zelevinsky K, Abing HK, Horvitz-Lennon M. Statistical Approaches for Quantifying the Quality of Neurosurgical Care. World Neurosurg. 2022;161:331-42.e1.

242.	Otieno GO, Hinako T, Motohiro A, Daisuke K, Keiko N. Measuring effectiveness of electronic medical records systems: towards building a composite index for benchmarking hospitals. Int J Med Inform. 2008;77(10):657-69.

243.	Oluwatola T, Saheed Dipo I, Omeje O, Oni F, Samuel OW, Sampson S, et al. Assessment of quality of maternal and newborn care and its determinants: a national study of primary health care facilities in Nigeria. BMC Health Services Research. 2025;25:1-12.

244.	Pakyz AL, Moczygemba LR, Wang H, Stevens MP, Edmond MB. An evaluation of the association between an antimicrobial stewardship score and antimicrobial usage. Journal of Antimicrobial Chemotherapy. 2014;70(5):1588-91.

245.	Pedroja AT, Blegen MA, Abravanel R, Stromberg AJ, Spurlock B. The relationship between hospital systems load and patient harm. J Patient Saf. 2014;10(3):168-75.

246.	Peters DH, Noor AA, Singh LP, Kakar FK, Hansen PM, Burnham G. A balanced scorecard for health services in Afghanistan. Bull World Health Organ. 2007;85(2):146-51.

247.	Popescu I, Cram P, Vaughan-Sarrazin MS. Differences in admitting hospital characteristics for black and white Medicare beneficiaries with acute myocardial infarction. Circulation. 2011;123(23):2710-6.

248.	Prinja S, Gupta R, Bahuguna P, Sharma A, Kumar Aggarwal A, Phogat A, et al. A composite indicator to measure universal health care coverage in India: way forward for post-2015 health system performance monitoring framework. Health Policy Plan. 2017;32(1):43-56.

249.	Profit J, Kowalkowski MA, Zupancic JA, Pietz K, Richardson P, Draper D, et al. Baby-MONITOR: a composite indicator of NICU quality. Pediatrics. 2014;134(1):74-82.

250.	Rhee JY, Garralda E, Namisango E, Luyirika E, de Lima L, Powell RA, et al. An Analysis of Palliative Care Development in Africa: A Ranking Based on Region-Specific Macroindicators. Journal of Pain & Symptom Management. 2018;56(2):230-8.

251.	Ross JS, Arling G, Ofner S, Roumie CL, Keyhani S, Williams LS, et al. Correlation of inpatient and outpatient measures of stroke care quality within veterans health administration hospitals. Stroke. 2011;42(8):2269-75.

252.	Ryan AM, Krinsky S, Maurer KA, Dimick JB. Changes in Hospital Quality Associated with Hospital Value-Based Purchasing. N Engl J Med. 2017;376(24):2358-66.

253.	Scaratti D, Calvo MC. Composite indicator to evaluate quality of municipal management of primary health care. Rev Saude Publica. 2012;46(3):446-55.

254.	Sequist TD, Schneider EC, Anastario M, Odigie EG, Marshall R, Rogers WH, et al. Quality monitoring of physicians: linking patients' experiences of care to clinical quality and outcomes. J Gen Intern Med. 2008;23(11):1784-90.

255.	Sequist TD, Von Glahn T, Li A, Rogers WH, Safran DG. Measuring chronic care delivery: patient experiences and clinical performance. Int J Qual Health Care. 2012;24(3):206-13.

256.	Shahian DM, Edwards FH, Ferraris VA, Haan CK, Rich JB, Normand SLT, et al. Quality Measurement in Adult Cardiac Surgery: Part 1-Conceptual Framework and Measure Selection. Annals of Thoracic Surgery. 2007;83(4 SUPPL.):S3-S12.

257.	Sharma A, Prinja S, Aggarwal AK. Comprehensive measurement of health system performance at district level in India: Generation of a composite index. Int J Health Plann Manage. 2019;34(4):e1783-e99.

258.	Shwartz M, Cohen AB, Restuccia JD, Ren ZJ, Labonte A, Theokary C, et al. How well can we identify the high-performing hospital? Med Care Res Rev. 2011;68(3):290-310.

259.	Silva AL, Mendes Ada C, Miranda GM, Sá DA, Souza WV, Lyra TM. Evaluation of maternal and neonatal hospital care: quality index of completeness. Rev Saude Publica. 2014;48(4):682-91; discussion 91.

260.	Staiger DO, Dimick JB, Baser O, Fan Z, Birkmeyer JD. Empirically derived composite measures of surgical performance. Med Care. 2009;47(2):226-33.

261.	Tardivo S, Moretti F, Agodi A, Appignanesi R, Baldovin T, Barchitta M, et al. Essential strategies in HAI prevention and control: performance assessment through the implementation of the HAI-CoSIP tool of the GISIO-SItI group. A pilot study in a sample of Italian Organizations. Ann Ig. 2018;30(5 Supple 2):70-85.

262.	Testa D, Andreano A, Zanfino A, Salvatori A, Milanese A, Mazzali C, et al. Development and user-oriented visualization of health care composite indicators at district level: territorial health profiles. Epidemiol Prev. 2024;48(6):406-18.

263.	Timbie JW, Shahian DM, Newhouse JP, Rosenthal MB, Normand SL. Composite measures for hospital quality using quality-adjusted life years. Stat Med. 2009;28(8):1238-54.

264.	Tsai TC, Orav EJ, Jha AK. Patient satisfaction and quality of surgical care in US hospitals. Ann Surg. 2015;261(1):2-8.

265.	Urick BY, Urmie JM. Framework for assessing pharmacy value. Res Social Adm Pharm. 2019;15(11):1326-37.

266.	van Doorn-Klomberg AL, Braspenning JC, Wolters RJ, Bouma M, de Grauw WJ, Wensing M. Organizational determinants of high-quality routine diabetes care. Scand J Prim Health Care. 2014;32(3):124-31.

267.	Vieira-Meyer A, Morais APP, Guimarães JMX, Campelo ILB, Vieira NFC, Machado M, et al. Infrastructure and work process in primary health care: PMAQ in Ceará. Rev Saude Publica. 2020;54:62.

268.	Weeks WB, Kotzbauer GR, Weinstein JN. Using publicly available data to construct a transparent measure of health care value: a method and initial results. The Milbank Quarterly. 2016;94(2):314-33.

269.	Weifeng W, Hess BJ, Lynn LA, Holmboe ES, Lipner RS. Measuring physicians' performance in clinical practice: reliability, classification accuracy, and validity. Eval Health Prof. 2010;33(3):302-20.

270.	Wilberforce M, Harrington V, Brand C, Tucker S, Abendstern M, Challis D. Towards integrated community mental health teams for older people in England: progress and new insights. Int J Geriatr Psychiatry. 2011;26(3):221-8.

271.	Wilhelm D, Lohmann J, De Allegri M, Chinkhumba J, Muula AS, Brenner S. Quality of maternal obstetric and neonatal care in low-income countries: development of a composite index. BMC Med Res Methodol. 2019;19(1):154.

272.	Yan LD, Mwale J, Straitz S, Biemba G, Bhutta Z, Ross JF, et al. Equity dimensions of the availability and quality of reproductive, maternal and neonatal health services in Zambia. Trop Med Int Health. 2018;23(4):433-45.

273.	Anhang Price R, Stucky B, Parast L, Elliott MN, Haas A, Bradley M, et al. Development of Valid and Reliable Measures of Patient and Family Experiences of Hospice Care for Public Reporting. J Palliat Med. 2018;21(7):924-32.

274.	Anhang Price R, Bradley MA, Ye F, Schlang D, DeYoreo M, Cleary PD, et al. Reliable and Valid Survey-Based Measures to Assess Quality of Care in Home-Based Serious Illness Programs. Journal of Palliative Medicine. 2022;25(6):864-72.

275.	Antoniotti S, Baumstarck-Barrau K, Siméoni MC, Sapin C, Labarère J, Gerbaud L, et al. Validation of a French hospitalized patients' satisfaction questionnaire: the QSH-45. Int J Qual Health Care. 2009;21(4):243-52.

276.	Asan O, Yu Z, Crotty BH. How clinician-patient communication affects trust in health information sources: Temporal trends from a national cross-sectional survey. PLoS One. 2021;16(2):e0247583.

277.	Berger BO, Strobino DM, Mehrtash H, Bohren MA, Adu-Bonsaffoh K, Leslie HH, et al. Development of measures for assessing mistreatment of women during facility-based childbirth based on labour observations. BMJ Glob Health. 2021;5(Suppl 2).

278.	Brown CM, Richards KM, Vohra Y, Kanu C, Stevens L, Sasane R, et al. Evaluation of access to care issues in patients with breast cancer. J Med Econ. 2021;24(1):38-45.

279.	Callegari LS, Mahorter SS, Benson SK, Zhao X, Schwarz EB, Borrero S. Perceived contraceptive counseling quality among veterans using VA primary care: Data from the ECUUN Study. Journal of General Internal Medicine. 2022;37(Suppl 3):698.

280.	Clarke-Deelder E, Eliakimu E, Mbatia R, Bohren MA, Mashasi I, Larson E. Measuring user experience of care among caregivers of sick children: Validation and descriptive analysis in a sample of 75 health facilities in rural Tanzania. Trop Med Int Health. 2022;27(3):317-29.

281.	Davis KK, Harris KG, Mahishi V, Bartholomew EG, Kenward K. Perceptions of Culture of Safety in Hemodialysis Centers. Nephrol Nurs J. 2016;43(2):119-26, 82; quiz 27.

282.	Desai AD, Zhou C, Simon TD, Mangione-Smith R, Britto MT. Validation of a Parent-Reported Hospital-to-Home Transition Experience Measure. Pediatrics. 2020;145(2).

283.	Dixon SK, Hoopes AJ, Benkeser D, Grigg A, Grow HM. Characterizing Key Components of a Medical Home Among Rural Adolescents. J Adolesc Health. 2016;58(2):141-7.

284.	Evensen CT, Yost KJ, Keller S, Arora NK, Frentzel E, Cowans T, et al. Development and Testing of the CAHPS Cancer Care Survey. J Oncol Pract. 2019;15(11):e969-e78.

285.	Gomez-Cano M, Lyratzopoulos G, Campbell JL, M NE, G AA. The underlying structure of the English Cancer Patient Experience Survey: Factor analysis to support survey reporting and design. Cancer Med. 2022;11(1):3-20.

286.	Hargraves JL, Hays RD, Cleary PD. Psychometric properties of the Consumer Assessment of Health Plans Study (CAHPS) 2.0 adult core survey. Health Serv Res. 2003;38(6 Pt 1):1509-27.

287.	Hotchkiss J. Hospice Glassdoor and CAHPS® Scores-Glassdoor Scores and Hospice Financial Characteristics Predict Hospice Consumer Assessment of Healthcare Providers and Systems Scores. Am J Hosp Palliat Care. 2022:10499091221099475.

288.	Jensen HI, Gerritsen RT, Koopmans M, Downey L, Engelberg RA, Curtis JR, et al. Satisfaction with quality of ICU care for patients and families: the euroQ2 project. Crit Care. 2017;21(1):239.

289.	Johnson MC, Jr., Liu H, Sorra J, Brock J, Gass B, Li J, et al. Development and psychometric properties of surveys to assess provider perspectives on the barriers and facilitators of effective care transitions. BMC Health Serv Res. 2021;21(1):478.

290.	Keller S, Col Martin GC, Evensen CT, Capt Mitton RH. The development and testing of a survey instrument for benchmarking dental plan performance: using insured patients' experiences as a gauge of dental care quality. J Am Dent Assoc. 2009;140(2):229-37.

291.	Kranz AM, Ryan J, Mahmud A, Setodji CM, Damberg CL, Timbie JW. Association of Primary and Specialty Care Integration on Physician Communication and Cancer Screening in Safety-Net Clinics. Prev Chronic Dis. 2020;17:E134.

292.	Langford A, Loeb S. Perceived Patient-Provider Communication Quality and Sociodemographic Factors Associated With Watching Health-Related Videos on YouTube: A Cross-Sectional Analysis. J Med Internet Res. 2019;21(5):e13512.

293.	Larson E, Hermosilla S, Kimweri A, Mbaruku GM, Kruk ME. Determinants of perceived quality of obstetric care in rural Tanzania: a cross-sectional study. BMC Health Serv Res. 2014;14:483.

294.	Lim MT, Ong SM, Tong SF, Groenewegen P, Sivasampu S. Comparison between primary care service delivery in Malaysia and other participating countries of the QUALICOPC project: a cross-sectional study. BMJ Open. 2021;11(5):e047126.

295.	Makary MA, Sexton JB, Freischlag JA, Millman EA, Pryor D, Holzmueller C, et al. Patient safety in surgery. Ann Surg. 2006;243(5):628-32; discussion 32-5.

296.	Milliken-Glabe SJ, Zuk J, Ziniel SI, Bjur KA, Alvarez M, Szolnoki JM, et al. First steps in validating the pediatric anesthesia parent satisfaction (PAPS) survey. Paediatr Anaesth. 2017;27(2):153-61.

297.	Minvielle E, Dervaux B, Retbi A, Aegerter P, Boumendil A, Jars-Guincestre MC, et al. Culture, organization, and management in intensive care: construction and validation of a multidimensional questionnaire. Journal of critical care. 2005;20(2):126-38.

298.	Olaisen RH, Flocke SA, Smyth KA, Schluchter MD, Koroukian SM, Stange KC. Developing a new measure of primary care using the Medical Expenditure Panel Survey. Medical care. 2019;57(6):475-81.

299.	O'Malley AJ, Zaslavsky AM, Hays RD, Hepner KA, Keller S, Cleary PD. Exploratory factor analyses of the CAHPS Hospital Pilot Survey responses across and within medical, surgical, and obstetric services. Health Serv Res. 2005;40(6 Pt 2):2078-95.

300.	Ragavan MI, Griffith KN, Cowden JD, Colvin JD, Bair-Merritt M. Parental Perceptions of Culturally Sensitive Care and Well-Child Visit Quality. Acad Pediatr. 2020;20(2):234-40.

301.	Reszel J, Weiss D, Darling EK, Sidney D, Van Wagner V, Soderstrom B, et al. Client Experience with the Ontario Birth Center Demonstration Project. J Midwifery Womens Health. 2021;66(2):174-84.

302.	Ruiz MA, Pardo A, Rejas J, Soto J, Villasante F, Aranguren JL. Development and validation of the "Treatment Satisfaction with Medicines Questionnaire" (SATMED-Q). Value Health. 2008;11(5):913-26.

303.	Sawicki GS, Garvey KC, Toomey SL, Williams KA, Chen Y, Hargraves JL, et al. Development and Validation of the Adolescent Assessment of Preparation for Transition: A Novel Patient Experience Measure. J Adolesc Health. 2015;57(3):282-7.

304.	Sequist TD, von Glahn T, Li A, Rogers WH, Safran DG. Statewide evaluation of measuring physician delivery of self-management support in chronic disease care. J Gen Intern Med. 2009;24(8):939-45.

305.	Solberg LI, Crain AL, Rubenstein L, Unützer J, Whitebird RR, Beck A. How much shared decision making occurs in usual primary care of depression? J Am Board Fam Med. 2014;27(2):199-208.

306.	Sorra J, Zebrak K, Carpenter D, Famolaro T, Rauch J, Li J, et al. Development and psychometric properties of surveys to assess patient and family caregiver experience with care transitions. BMC Health Serv Res. 2021;21(1):785.

307.	Sorra J, Zebrak K, Yount N, Famolaro T, Gray L, Franklin M, et al. Development and pilot testing of survey items to assess the culture of value and efficiency in hospitals and medical offices. BMJ Qual Saf. 2022;31(7):493-502.

308.	Teruya C, Joshi V, Urada D, Trabin T, Iturrios-Fourzan I, Huang Y-C. Development and Measurement of the Treatment Perceptions Survey (TPS) for Clients with Substance Use Disorders. Journal of Behavioral Health Services & Research. 2022;49(2):190-203.

309.	Toomey SL, Zaslavsky AM, Elliott MN, Gallagher PM, Fowler FJ, Jr., Klein DJ, et al. The Development of a Pediatric Inpatient Experience of Care Measure: Child HCAHPS. Pediatrics. 2015;136(2):360-9.

310.	Tuladhar S, Delius M, Siebeck M, Oberhauser C, Paudel D, Rehfuess E. Standards of care and determinants of women’s satisfaction with delivery services in Nepal: a multi-perspective analysis using data from a health facility-based survey. BMC Pregnancy and Childbirth. 2024;24(1):132.

311.	Wahl H. Measuring Patient Experience in Hospital Maternity Care. 2020.

312.	Weidmer BA, Brach C, Hays RD. Development and evaluation of CAHPS survey items assessing how well healthcare providers address health literacy. Med Care. 2012;50(9 Suppl 2):S3-11.

313.	Wiegers TA. The quality of maternity care services as experienced by women in the Netherlands. BMC Pregnancy Childbirth. 2009;9:18.

314.	Wojcik BM, McKinley SK, Amari N, Chang DC, Wachtel H, Petrusa E, et al. A comparison of patient satisfaction when office-based procedures are performed by general surgery residents versus an attending surgeon. Surgery. 2019;166(1):116-22.

315.	Ye F, Parast L, Hays RD, Elliott MN, Becker K, Lehrman WG, et al. Development and validation of a patient experience of care survey for emergency departments. Health Serv Res. 2022;57(1):102-12.

316.	Zebrak K, Yount N, Sorra J, Famolaro T, Gray L, Carpenter D, et al. Development, Pilot Study, and Psychometric Analysis of the AHRQ Surveys on Patient Safety Culture™ (SOPS(®)) Workplace Safety Supplemental Items for Hospitals. Int J Environ Res Public Health. 2022;19(11).

317.	Amadeo B, Dumartin C, Parneix P, Fourrier-Réglat A, Rogues AM. Relationship between antibiotic consumption and antibiotic policy: an adjusted analysis in the French healthcare system. J Antimicrob Chemother. 2011;66(2):434-42.

318.	Aggarwal RK, Singh D. Adherence to Quality in District Hospitals of Haryana: A Recent Assessment as per Indian Public Health Standards (IPHS). Journal of Health Management. 2023;25(4):987-95.

319.	Das T, Das D. Does the augmentation of monetary and non-monetary factors prerequisite for the improvement of health outcomes? Evidence from the Indian states. Int J Health Plann Manage. 2022;37(2):1131-56.

320.	Gupta A, Chen Q, Wilson LE, Huang B, Pisu M, Liang M, et al. Factor Analysis of Health Care Access With Ovarian Cancer Surgery and Gynecologic Oncologist Consultation. JAMA Netw Open. 2023;6(2):e2254595.

321.	Henry EG, Semrau K, Hamer DH, Vian T, Nambao M, Mataka K, et al. The influence of quality maternity waiting homes on utilization of facilities for delivery in rural Zambia. Reprod Health. 2017;14(1):68.

322.	Iyer HS, Kamanzi E, Mugunga JC, Finnegan K, Uwingabiye A, Shyaka E, et al. Improving district facility readiness: a 12-month evaluation of a data-driven health systems strengthening intervention in rural Rwanda. Glob Health Action. 2015;8:28365.

323.	Pelly L, Srivastava K, Singh D, Anis P, Mhadeshwar VB, Kumar R, et al. Readiness to provide child health services in rural Uttar Pradesh, India: mapping, monitoring and ongoing supportive supervision. BMC Health Services Research. 2021;21(1):1-11.

324.	Powell-Jackson T, Acharya A, Mills A. An assessment of the quality of primary health care in India. Economic and Political Weekly. 2013:53-61.

325.	Reddy BP, O'Neill S, O'Neill C. Developing composite indices of geographical access and need for nursing home care in Ireland using multiple criteria decision analysis. HRB Open Res. 2020;3:65.

326.	Shin DW, Hwang SS, Oh J, Kim JH, Park JH, Cho J, et al. Variations in pain management outcomes among palliative care centers and the impact of organizational factors. Cancer. 2012;118(22):5688-97.
