# Supplementary material for: Methods to derive composite indicators used for quality and safety measurement and monitoring in healthcare: a scoping review
Source: Front Health Serv. 2026 Aug 4;6:1852718. doi: 10.3389/frhs.2026.1852718 (PMC13481381; doi:10.3389/frhs.2026.1852718)
Supplement: Supplementary file 3 [file Supplementaryfile3.docx]

| **Supplementary File 3**  **List of Methodology Publications** | | |
| --- | --- | --- |
| **Ref** | **Lead Author & Year** | **Country** |
| **(**1) | Adelman 2020 | United States |
| (2) | AHRQ 2012 | United States |
| (3) | Austin 2019 | Non-specific |
| (4) | Barclay 2019 | Non-specific |
| (5) | Barclay 2022 | United States |
| (6) | Bhattacharyya 2009 | United States |
| (7) | Bilimoria 2016 | United States |
| (8) | Bilimoria 2021 | United States |
| (9) | Blay 2019 | United States |
| (10) | Brown 2006 | UK |
| (11) | Castellucci 2018 | United States |
| (12) | Castellucci 2018 | United States |
| (13) | Castellucci 2019 | United States |
| (14) | Chen 2016 | United States |
| (15) | Chung 2019 | United States |
| (16) | Couralet 2011 | Other: France |
| (17) | deJager 2023 | United States |
| (18) | Dervaux 2004 | United States |
| (19) | Eapen 2011 | United States |
| (20) | Emmert 2024 | Other: Germany |
| (21) | Fontana 2019 | United States |
| (22) | Friebel 2019 | Non-specific |
| (23) | Garcia-Lorenzo 2024 | Other: Spain |
| (24) | Gettel 2024 | United States |
| (25) | Glance 2020 | United States |
| (26) | Glickman 2009 | United States |
| (27) | Goddard 2010 | Non-specific |
| (28) | Jacobs 2004 | UK |
| (29) | Jacobs 2005 | UK |
| (30) | Kara 2022 | Non-specific |
| (31) | Khayal 2023 | United States |
| (32) | Kurian 2021 | United States |
| (33) | Liang 2018 | United States |
| (34) | Mannion 2005 | UK |
| (35) | Martsolf 2013 | United States |
| (36) | McKenna 2020 | Non-specific |
| (37) | Moore 2013 | Canada |
| (38) | Mukamel 2020 | United States |
| (39) | Mukherjee 2013 | United States |
| (40) | NationalQualityForum 2013 | United States |
| (41) | Nerenz 2020 | United States |
| (42) | Normand 2008 | United States |
| (43) | O'Brien 2007 | United States |
| (44) | Peterson 2010 | United States |
| (45) | Reeves 2007 | UK |
| (46) | Rohrbacher 2025 | Other: Germany |
| (47) | Salerno 2025 | United States |
| (48) | Schang 2016 | UK |
| (49) | Schoner 2023 | Other: Germany |
| (50) | Shahian 2022 | United States |
| (51) | Shwartz 2008 | United States |
| (52) | Shwartz 2009 | United States |
| (53) | Shwartz 2013 | United States |
| (54) | Shwartz 2015 | United States |
| (55) | Shwartz 2016 | United States |
| (56) | Shwartz 2017 | United States |
| (57) | Simms 2013 | UK |
| (58) | Snelling 2003 | UK |
| (59) | Steele 2021 | United States |
| (60) | Teixeira-Pinto 2008 | United States |
| (61) | vanDoorn-Klomberg 2013 | Other: The Netherlands |
| (62) | Vara 2025 | Other: Portugal |
| (63) | Vidoli 2024 | Other: Italy |
| (64) | Weimer 2019 | United States |
| (65) | Zimlich 2017 | United States |

**REFERENCES:**

1.	Adelman D. An Efficient Frontier Approach to Scoring and Ranking Hospital Performance. Operations Research. 2020;68(3):762.

2.	Agency for Healthcare Research and Quality. Inpatient Quality Indicators Composite Measure Workgroup Final Report. 2012.

3.	Austin PC, Ceyisakar IE, Steyerberg EW, Lingsma HF, Marang-van de Mheen PJ. Ranking hospital performance based on individual indicators: can we increase reliability by creating composite indicators? BMC Med Res Methodol. 2019;19(1):131.

4.	Barclay M, Dixon-Woods M, Lyratzopoulos G. The problem with composite indicators. BMJ Qual Saf. 2019;28(4):338-44.

5.	Barclay ME, Dixon-Woods M, Lyratzopoulos G. Concordance of Hospital Ranks and Category Ratings Using the Current Technical Specification of US Hospital Star Ratings and Reasonable Alternative Specifications. JAMA Health Forum. 2022;3(5):e221006.

6.	Bhattacharyya T, Freiberg AA, Mehta P, Katz JN, Ferris T. Measuring the report card: the validity of pay-for-performance metrics in orthopedic surgery. Health Aff (Millwood). 2009;28(2):526-32.

7.	Bilimoria KY, Barnard C. The New CMS Hospital Quality Star Ratings: The Stars Are Not Aligned. Jama. 2016;316(17):1761-2.

8.	Bilimoria KY, Barnard C. An Evolving Hospital Quality Star Rating System From CMS: Aligning the Stars. Jama. 2021;325(21):2151-2.

9.	Blay E, Jr., Huang R, Chung JW, Yang AD, DeLancey JO, Merkow RP, et al. Evaluating the Impact of the Venous Thromboembolism Outcome Measure on the PSI 90 Composite Quality Metric. Jt Comm J Qual Patient Saf. 2019;45(3):148-55.

10.	Brown C, Lilford R. Cross sectional study of performance indicators for English Primary Care Trusts: testing construct validity and identifying explanatory variables. BMC Health Serv Res. 2006;6:81.

11.	Castellucci M. CMS planning modest changes to hospital star-rating system. Modern Healthcare. 2018;48(28):10.

12.	Castellucci M. CMS hospital star ratings were wrong from the start, health system finds. Modern Healthcare. 2018;48(25):12.

13.	Castellucci M. Hospitals hopeful big changes are coming to the CMS' star ratings. Modern Healthcare. 2019;49(9):6.

14.	Chen Q, Rosen AK, Borzecki A, Shwartz M. Using Harm-Based Weights for the AHRQ Patient Safety for Selected Indicators Composite (PSI-90): Does It Affect Assessment of Hospital Performance and Financial Penalties in Veterans Health Administration Hospitals? Health Serv Res. 2016;51(6):2140-57.

15.	Chung JW, Dahlke AR, Barnard C, DeLancey JO, Merkow RP, Bilimoria KY. The Centers For Medicare And Medicaid Services Hospital Ratings: Pitfalls Of Grading On A Single Curve. Health Aff (Millwood). 2019;38(9):1523-9.

16.	Couralet M, Guérin S, Le Vaillant M, Loirat P, Minvielle E. Constructing a composite quality score for the care of acute myocardial infarction patients at discharge: impact on hospital ranking. Med Care. 2011;49(6):569-76.

17.	de Jager E, Levine AA, Sidey-Gibbons CJ, Udyavar NR, Liu C, Lamaina M, et al. Development and Validation of a Novel Literature-Based Method to Identify Disparity-Sensitive Surgical Quality Metrics. J Am Coll Surg. 2023;237(6):856-61.

18.	Dervaux B, Leleu H, Valdmanis V. Estimating tradeoffs among health care system's objectives. Health Services and Outcomes Research Methodology. 2004;5(1):39-58.

19.	Eapen ZJ, Fonarow GC, Dai D, O'Brien SM, Schwamm LH, Cannon CP, et al. Comparison of composite measure methodologies for rewarding quality of care: an analysis from the American Heart Association's Get With The Guidelines program. Circ Cardiovasc Qual Outcomes. 2011;4(6):610-8.

20.	Emmert M, Rohrbacher S, Meier F, Heppe L, Drach C, Schindler A, et al. The elicitation of patient and physician preferences for calculating consumer-based composite measures on hospital report cards: results of two discrete choice experiments. Eur J Health Econ. 2024;25(6):1071-85.

21.	Fontana MA, Lyman S, Islam W, MacLean CH. When Stars Do Not Align: Overall Hospital Quality Star Ratings and the Volume-Outcome Association. JB JS Open Access. 2019;4(1):e0044.

22.	Friebel R, Steventon A. Composite measures of healthcare quality: sensible in theory, problematic in practice. BMJ Qual Saf. 2019;28(2):85-8.

23.	García-Lorenzo B, Alayo I, Arrospide A, Gorostiza A, Fullaondo A. Disentangling the value equation: a step forward in value-based healthcare. Eur J Public Health. 2024;34(4):632-8.

24.	Gettel CJ, Bagshaw K, Qin L, Lin Z, Rothenberg E, Omotosho P, et al. Calculation of Overall Hospital Quality Star Ratings With and Without Inclusion of the Peer Grouping Step. JAMA Netw Open. 2024;7(5):e2411933.

25.	Glance LG, Thirukumaran CP, Li Y, Gao S, Dick AW. Improving The Accuracy Of Hospital Quality Ratings By Focusing On The Association Between Volume And Outcome. Health Aff (Millwood). 2020;39(5):862-70.

26.	Glickman SW, Boulding W, Roos JM, Staelin R, Peterson ED, Schulman KA. Alternative pay-for-performance scoring methods: implications for quality improvement and patient outcomes. Med Care. 2009;47(10):1062-8.

27.	Goddard M, Jacobs R. Using composite indicators to measure performance in health care. Smith PC ME, Papanicolas I, Leatherman S,, editor: Cambridge University Press; 2010.

28.	Jacobs R, Smith P, Goddard M. Measuring performance: an examination of composite performance indicators. 2004.

29.	Jacobs R, Goddard M, Smith PC. How robust are hospital ranks based on composite performance measures? Med Care. 2005;43(12):1177-84.

30.	Kara P, Valentin JB, Mainz J, Johnsen SP. Composite measures of quality of health care: Evidence mapping of methodology and reporting. PLoS ONE. 2022;17(5 May).

31.	Khayal IS, Sanz JT. From measures to action: can integrating quality measures provide system-wide insights for quality improvement decision making? BMJ Health Care Inform. 2023;30(1).

32.	Kurian N, Maid J, Mitra S, Rhyne L, Korvink M, Gunn LH. Predicting Hospital Overall Quality Star Ratings in the USA. Healthcare (Basel). 2021;9(4).

33.	Liang S, Luketich JD, Sarkaria IS. A perspective on the Society of Thoracic Surgeons Composite Score for evaluating esophagectomy for esophageal cancer. Journal of Thoracic Disease. 2018;10(1):94-7.

34.	Mannion R, Davies H, Marshall M. Impact of star performance ratings in English acute hospital trusts. J Health Serv Res Policy. 2005;10(1):18-24.

35.	Martsolf GR, Scanlon DP, Christianson JB. Multistakeholder perspectives on composite measures of ambulatory care quality: a qualitative descriptive study. Med Care Res Rev. 2013;70(4):434-48.

36.	McKenna SP, Heaney A. Composite outcome measurement in clinical research: the triumph of illusion over reality? J Med Econ. 2020;23(10):1196-204.

37.	Moore L, Lavoie A, Sirois MJ, Belcaid A, Bourgeois G, Lapointe J, et al. A comparison of methods to obtain a composite performance indicator for evaluating clinical processes in trauma care. J Trauma Acute Care Surg. 2013;74(5):1344-50.

38.	Mukamel DB, Weimer DL, Shi Y, Ladd H, Saliba D. Comparison of Consumer Rankings With Centers for Medicare & Medicaid Services Five-Star Rankings of Nursing Homes. JAMA Netw Open. 2020;3(5):e204798.

39.	Mukherjee S, Rodriguez HP, Elliott MN, Crane PK. Modern psychometric methods for estimating physician performance on the Clinician and Group CAHPS survey. Health Services & Outcomes Research Methodology. 2013;13(2-4):109-23.

40.	National Quality Forum. Composite performance measure evaluation guidance. 2013.

41.	Nerenz DR, Hu J, Waterman B, Jordan J. Weighting of Measures in the Safety of Care Group of the Overall Hospital Quality Star Rating Program: An Alternative Approach. American journal of medical quality : the official journal of the American College of Medical Quality. 2020;35(1):46-51.

42.	Normand SLT, Wolf RE, McNeil BJ. Discriminating quality of hospital care in the United States. Medical Decision Making. 2008;28(3):308-22.

43.	O'Brien SM, DeLong ER, Dokholyan RS, Edwards FH, Peterson ED. Exploring the behavior of hospital composite performance measures: an example from coronary artery bypass surgery. Circulation. 2007;116(25):2969-75.

44.	Peterson ED, DeLong ER, Masoudi FA, O'Brien SM, Peterson PN, Rumsfeld JS, et al. ACCF/AHA 2010 Position Statement on Composite Measures for Healthcare Performance Assessment: a report of American College of Cardiology Foundation/American Heart Association Task Force on Performance Measures (Writing Committee to Develop a Position Statement on Composite Measures). J Am Coll Cardiol. 2010;55(16):1755-66.

45.	Reeves D, Campbell SM, Adams J, Shekelle PG, Kontopantelis E, Roland MO. Combining multiple indicators of clinical quality: an evaluation of different analytic approaches. Med Care. 2007;45(6):489-96.

46.	Rohrbacher S, Emmert M. The elicitation of patient preferences for hip replacement surgery: a discrete choice experiment. BMC Health Serv Res. 2025;25(1):268.

47.	Salerno S, Yang E, Dahlerus C, Hirth RA, Han P, Xu T, et al. Adding New Components to a Composite Quality Metric: How Good Is Good Enough? Med Care. 2025;63(4):293-9.

48.	Schang L, Hynninen Y, Morton A, Salo A. Developing robust composite measures of healthcare quality - Ranking intervals and dominance relations for Scottish Health Boards. Soc Sci Med. 2016;162:59-67.

49.	Schöner L, Kuklinski D, Geissler A, Busse R, Pross C. A composite measure for patient-reported outcomes in orthopedic care: design principles and validity checks. Qual Life Res. 2023;32(8):2341-51.

50.	Shahian DM, Bowdish ME, Bloom JP, Wyler von Ballmoos MC, Edgerton JR, Antman MS, et al. The Society of Thoracic Surgeons Coronary Artery Bypass Graft Composite Measure: 2021 Methodology Update. Ann Thorac Surg. 2022;113(6):1954-61.

51.	Shwartz M, Ren J, Peköz EA, Wang X, Cohen AB, Restuccia JD. Estimating a composite measure of hospital quality from the Hospital Compare database: differences when using a Bayesian hierarchical latent variable model versus denominator-based weights. Med Care. 2008;46(8):778-85.

52.	Shwartz M, Burgess J, Berlowitz D. Benefit-of-the-doubt approaches for calculating a composite measure of quality. Health Services & Outcomes Research Methodology. 2009;9(4):234-51.

53.	Shwartz M, Peköz EA, Christiansen CL, Burgess JF, Berlowitz D. Shrinkage Estimators for a Composite Measure of Quality Conceptualized as a Formative Construct. Health Services Research. 2013;48(1):271-89.

54.	Shwartz M, Restuccia JD, Rosen AK. Composite Measures of Health Care Provider Performance: A Description of Approaches. Milbank Q. 2015;93(4):788-825.

55.	Shwartz M, Burgess JF, Jr., Zhu J. A DEA based composite measure of quality and its associated data uncertainty interval for health care provider profiling and pay-for-performance. European Journal of Operational Research. 2016;253(2):489.

56.	Shwartz M, Rosen AK, Burgess JF, Jr. Can Composite Measures Provide a Different Perspective on Provider Performance Than Individual Measures? Med Care. 2017;55(12):e99-e103.

57.	Simms AD, Batin PD, Weston CF, Fox KA, Timmis A, Long WR, et al. An evaluation of composite indicators of hospital acute myocardial infarction care: a study of 136,392 patients from the Myocardial Ischaemia National Audit Project. Int J Cardiol. 2013;170(1):81-7.

58.	Snelling I. Do star ratings really reflect hospital performance? J Health Organ Manag. 2003;17(3):210-23.

59.	Steele R, Ford C, Niles B, Coffin J. 2021 Hospital Quality Star Ratings: How Public Input Pressured Reform in Hospital Compare Star Ratings Calculation. Journal of Medical Practice Management. 2021;37(3):103-5.

60.	Teixeira-Pinto A, Normand SL. Statistical methodology for classifying units on the basis of multiple-related measures. Stat Med. 2008;27(9):1329-50.

61.	van Doorn-Klomberg AL, Braspenning JC, Feskens RC, Bouma M, Campbell SM, Reeves D. Precision of individual and composite performance scores: the ideal number of indicators in an indicator set. Med Care. 2013;51(1):115-21.

62.	Vara GM, Gomes MC, Ferreira DC. Assessing the performance of Portuguese public hospitals before and during COVID-19 outbreak, with optimistic and pessimistic benchmarking approaches. Health Care Manag Sci. 2025;28(1):1-27.

63.	Vidoli F, Pignataro G, Battiato S, Guarnera F, Guccio C. One for all? Assessing the quality of Italian hospital care with the "benefit of the doubt" composite indicator methods. Health Econ Rev. 2024;14(1):83.

64.	Weimer DL, Saliba D, Ladd H, Shi Y, Mukamel DB. Using contingent valuation to develop consumer-based weights for health quality report cards. Health Serv Res. 2019;54(4):947-56.

65.	Zimlich RRN. Hospital star ratings criticized for bias, incomplete data. Managed Healthcare Executive. 2017;27(5):8-10.
